# ML-Driven optimization of two-phase microfluidic cooling using acoustofluidic bubble actuation and nanoarray-coated micropin structures

**DOI:** 10.1038/s41598-025-23871-6

**Published:** 2025-11-17

**Authors:** Seyed Hamed Godasiaei, Pouyan Talebizadehsardari, Amir Keshmiri

**Affiliations:** 1 School of Chemical Engineering and Technology, Xi’an Jiaotong University, Suzhou, PR China; 2 Power Electronics, Machines and Control (PEMC) Institute, University of Nottingham, Nottingham, UK; 3 Research & Development Team, Manchester Simulations Limited (Mansim), Manchester, UK; 4 School of Engineering, University of Manchester, Manchester, UK

**Keywords:** Two-Phase cooling, Micropin structures, Acoustofluidic, Critical heat flux, Statistical methods, Machine learning., Engineering, Materials science, Mathematics and computing, Nanoscience and technology, Physics

## Abstract

This study presents a novel two-phase microfluidic cooling strategy integrating acoustofluidic bubble activation, nanoarray-coated micropin structures, and machine learning-guided optimization. Unlike conventional passive cooling, ultrasound-driven bubble actuation stabilizes boiling, prevents drying, and ensures uniform heat distribution, while nanoarray-coated micropins enhance liquid refilling via capillary forces and improve surface wettability. Experimental data were analyzed using deep neural networks (DNN), long short-term memory (LSTM) models, and statistical correlation methods (Spearman and Kendall), with interpretability provided by SHAP (DeepSHAP) and partial dependence plots (PDP). A comparison of model performance revealed that the LSTM achieved lower prediction errors than the DNN across all evaluated metrics (MAE 0.0055, SMAPE (0.8), and RMSE 0.0072), indicating its outperforming performance. SHAP and statistical analyses identified initial temperature as the most influential factor affecting heat transfer coefficient (HTC), followed by chipset material (S30-120 and stainless steel). Secondary factors include chipset configuration (SS, S30-120, S-nanorod, S-nanosheet) and nanoparticle type (SiO₂, ZnO), which significantly modulate bubble dynamics and thermal performance. PDP results highlight acoustofluidic excitation as the primary positive contributor, while flow rate and nanostructured surfaces provide moderate enhancements.

**Supplementary Information:**

The online version contains supplementary material available at 10.1038/s41598-025-23871-6.

## Introduction

In an era defined by the pervasive influence of high-powered electronic systems across diverse domains, ranging from spacecraft and radar installations to integrated electronics, data centers, and fast-charging batteries, the significance of efficient thermal management cannot be overstated^1^. The ongoing integration and miniaturization of power devices intensifies the need for effective heat dissipation mechanisms^2,3^. Among the myriad strategies deployed to address this pressing challenge, the utilization of the liquid-gas phase change process has emerged as a standout contender^4,5^. Leveraging the latent heat of vaporization, this process offers a potent means of facilitating efficient heat transfer, holding the promise of optimal performance, reliability, and safety^6^. However, despite the inherent advantages conferred by the liquid-gas phase change process, its efficacy encounters a fundamental constraint in the form of thermal resistance^7^. This resistance poses a significant obstacle to efficient heat extraction and typically manifests between the heat source, often symbolized by a semiconductor chip, and its surrounding packaging^8,9^. To surmount this limitation and bolster the heat extraction capabilities, the integration of micrometer-sized structures or channels directly onto the heat source or adjacent components has emerged as a tantalizing solution^10,11^. By augmenting the available surface area for heat transfer, these microstructures enable more efficient dissipation of thermal energy^12,13^. Moreover, the presence of microchannels serves to facilitate the boiling process, thereby enhancing overall heat transfer performance^14^. This advancement not only addresses the critical challenge of thermal resistance but also has the potential to redefine thermal management strategies for high-power electronic systems^15,16^.

Enhancing cooling efficiency in microchannels, particularly those in direct contact with heat sources like chips, is crucial across various industries^17,18^. This endeavor involves overcoming challenges and leveraging emerging opportunities through technological advancements and innovative research methodologies^19^. One approach to improving cooling efficiency involves extending macroscale structures such as ribs and fins to increase the surface area for heat dissipation^20^. Additionally, efforts to enhance nucleation and disruption or mixing through external fields like electric fields have been explored to optimize heat transfer processes in microchannels^21^. In a recent study by Huang et al.^22^, researchers investigated a radial micropin heatsink designed to dissipate heat from high heat flux areas or hot spots. Their systematic testing examined factors such as saturation temperature, inlet conditions, and heat flux, revealing the heat sink’s ability to maintain stable and efficient heat transfer performance under various conditions, indicating its potential for high heat flux applications. Bandari et al.^23^ conducted a comprehensive analysis of micropin, exploring factors like density, arrangement, and cross-sectional shape. Their findings highlighted the significant impact of downstream vortices on the thermo-hydraulic performance of micropin, crucial for optimizing microchannel cooling systems. Similarly, Radmerd et al.^24^ focused on heat transfer from a micropin cooler attached to a silicon chip. Employing advanced techniques like artificial neural networks combined with NSGA-II optimization, they improved the cooling device’s thermal and hydraulic performance, demonstrating the potential of computational approaches in optimizing microchannel cooling systems. Zhu et al.^25^ investigated the heat transfer coefficient (HTC) of welding flow in small channels with micropin using a machine learning model. Their results showcased the model’s high predictive accuracy, underscoring its potential for predicting and optimizing heat transfer processes in microchannels. Despite these advancements, challenges persist in achieving simultaneous improvements in all heat transfer properties in microchannels^26,27^. Critical parameters like critical heat flux (CHF) and HTC pose challenges, as enhancing one often compromises the other^28,29^. To address these challenges, microfluidic technology offers precise control over nanomaterial structures in confined spaces, with passive and active methods for enhancing mixing^30,31^. Passive methods involve perturbing flow fields, while active techniques like acoustic microfluidics disrupt laminar flows using acoustic waves, promising improved mass transfer and mixing^30,32^. While progress has been made in understanding and optimizing heat transfer processes in microchannels, further research is needed to explore passive and active methods fully, driving innovation and discovery in this field.

This study addresses two exciting areas with the aim of advancing machine learning-assisted thermal management techniques. First, it focuses on the production of silica nanofluid microfluids tailored for highly efficient two-phase cooling using a micropin structure. This innovative approach leverages microfluidic techniques to engineer nanofluids with improved thermal properties, specifically designed for optimal performance in two-phase cooling applications. By incorporating a micropin structure, the heat transfer capability of these nanofluids is enhanced, making them suitable for advanced cooling systems. Second, this study investigates micropin surfaces coated with functional nanoarray coatings and uses acoustofluidic bubble assistance for efficient cooling of a liquid-vapor phase change chip. This method uses acoustofluidic principles to enhance heat dissipation and offers a promising solution for chip cooling applications. By functionalizing the nanoarrays and coating the micropin surfaces, the liquid-vapor phase change cooling efficiency is significantly improved. Experimental data–including volume fraction, velocity, temperature, heat flux, ZnO nanoparticles, silicon dioxide, S30-120 chipset, SS chipset, S-nanorod chipset, S-nanoplate chipset, and acoustofluidic parameters–are analyzed using deep neural networks (DNN), long short-term memory models (LSTM), and statistical methods (Spearman and Kendall) to avoid biases and account for pre-processing. Their interpretation is provided through SHAP (G-DeepSHAP) and partial dependence plots (PDP). All statistical analyses and machine learning are implemented in Python, allowing for reproducible, data-driven optimization of nanofluid design and acoustofluidic-assisted cooling performance.

## Methods and materials

### Problem description

**Fig. 1:**
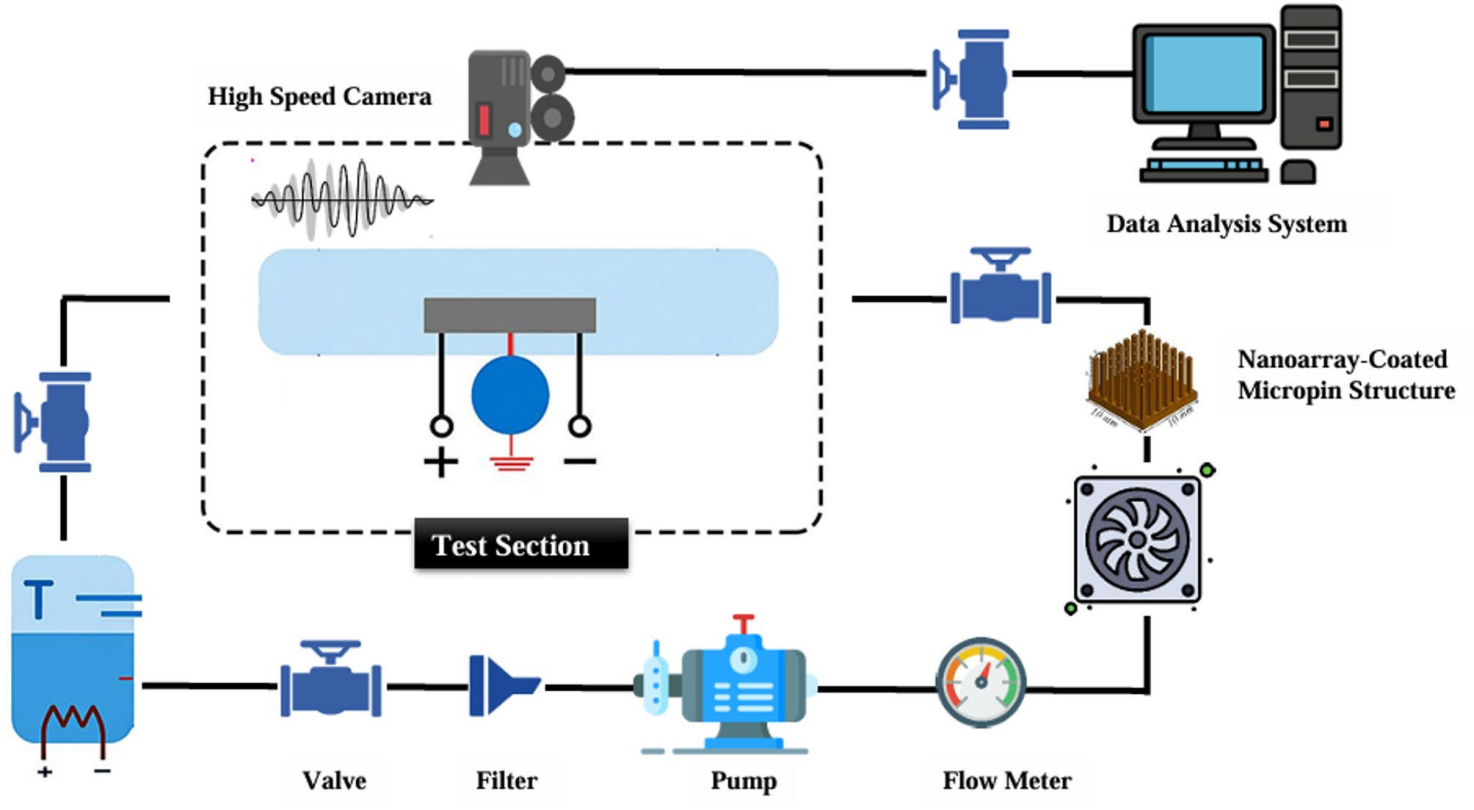
Schematic of a two-phase microfluidic cooling system with acoustofluidic bubble actuation and ML-Based.

Figure 1 shows a schematic of a two-phase microfluidic cooling system integrated with machine learning (ML) that uniquely combines acoustofluidic bubble actuation, nanoparticle-enhanced coolants, and nanoarray-coated micropin structures to enable advanced thermal management of high-power electronic devices. The cycle begins in a fluid reservoir containing either a base coolant or a nanoparticle-modified nanofluid, where a heating element precisely controls the inlet temperature to establish constant and reproducible boundary conditions prior to circulation. A miniature pump then drives the coolant through a closed loop, with flow control valves regulating the mass flow rate and pressure sensors ensuring hydraulic stability and maintaining a robust two-phase flow regime. The fluid subsequently enters the acoustofluidic chamber, where a piezoelectric transducer, in conjunction with embedded electrodes, generates high-frequency ultrasound waves that stimulate bubble nucleation and dynamics in a deterministic manner. Unlike conventional pool or flow boiling, where bubble formation is stochastic and often leads to localized dry-out spots, temperature oscillations, and non-uniform heat fluxes, this acoustically assisted excitation modulates nucleation frequency, accelerates bubble detachment from heated surfaces, and enhances liquid replenishment, thereby delaying the onset of critical heat flux (CHF) and stabilizing the boiling process under high thermal loads. After undergoing this bubble conditioning step, the coolant passes through the micropin-fin array, whose enlarged surface area and tailored geometry provide improved heat dissipation pathways. Functional nanoarray coatings–engineered in the form of nanorods and nanosheets–are deposited on the micropin surfaces to further improve surface wettability, enhance capillary-driven liquid return, and increase the density of active nucleation sites. These nanoengineered features not only intensify phase-change heat transfer but also ensure uniform thermal distribution across the chip, addressing hot-spot formation that limits the reliability of advanced electronics. Downstream, a compressed air fan accelerates vapor condensation and reinforces liquid recirculation, maintaining steady-state operation and preventing overheating during continuous high-power operation. Throughout the cooling cycle, a distributed network of sensors records real-time temperature, pressure, and flow rate data, while a high-speed camera provides visual diagnostics of bubble nucleation, growth, coalescence, and departure within the microchannel. These experimental datasets are continuously processed through a Python-based ML framework that performs statistical and predictive analyses while autonomously tuning critical operating parameters–including pump speed, valve positions, acoustic frequency and amplitude, and fan velocity–to dynamically optimize cooling performance and energy efficiency. The experimental core consists of a cover plate, channel plate, and support plate enclosing a microchannel of 51.5 × 10 × 1 mm, within which different chip surfaces (S30-120, stainless steel, S-nanorod, and S-nanosheet) were systematically tested. In parallel, the coolant composition was varied by dispersing SiO₂ and ZnO nanoparticles in volume fractions from 0 to 0.05 to quantify the role of nanofluids in augmenting nucleate boiling, thermal conductivity, and surface rewetting properties. Taken together, this integrated framework demonstrates how acoustically controlled bubble dynamics, nanoengineered pin-fin arrays, nanoparticle-enhanced fluids, and machine learning-based optimization synergistically interact to deliver superior two-phase cooling, minimize pumping and energy demands, and significantly improve the reliability of thermal management systems compared to conventional passive cooling approaches.

### Effective techniques for data collection, feature extraction, and information preparation

This study adopts a multidisciplinary approach to investigate high-efficiency two-phase cooling, with a focus on innovative micropin structures and the application of ML methodologies. Advanced algorithms such as Long Short-Term Memory (LSTM) and Deep Neural Networks (DNN) are utilized to explore the complexities of this cooling technique. Central to this research is a carefully curated dataset containing 598 rows of experimental data encompassing 11 input variables and one output variable, representing 7,176 individual data points, which collectively represent key factors influencing cooling efficiency^33,34^. A detailed overview of these variables and their interdependencies is presented in Table 1, while Fig. 4 illustrates the data split, with 80% allocated to training and 20% reserved for testing to ensure robust analysis. To maintain consistency and objectivity, the study employs strict protocols during the training and testing phases. These are supported by the “random mode” functionality provided by the scikit-learn package in Python, which ensures uniformity in the selection of data samples. The integration of structured datasets and machine learning methods places this research at the forefront of addressing the challenges associated with high-efficiency two-phase cooling for novel micropin structures. The ML models used in this work are implemented using the scikit-learn package, which is well-known for its adaptability and dependability. Its extensive toolkit makes data analysis, modeling, and visualization easier while guaranteeing fair and accurate assessments of all created models. By leveraging these capabilities, the research delves deeply into the intricate dynamics of two-phase cooling, aiming to provide actionable insights for industries where efficient heat transfer is critical. This pioneering study not only enhances understanding of high-efficiency two-phase cooling but also contributes strategies to advance real-world thermal management applications. By combining machine learning methodologies with structured datasets, it underscores the potential of innovative approaches to optimize cooling performance in novel micropin structures.

**Table 1:** Features of the dataset created for this study’s input and output.

No.	Description	Units	Parameter	Input and output
1	Volume fraction	*%*	*vf*	Input
2	Velocity	*m/s*	*u*	Input
3	Temperature	*°C*	*T*	Input
4	Heat flux	*W/m* ^*2*^	*Q*	Input
5	ZnO nanoparticle	*-*	*ZnO*	Input
6	Silicon dioxide	*-*	*SiO*_*2*_	Input
7	S30-120 chipset	*-*	S30-120	Input
8	SS chipset	*-*	*SS*	Input
9	S-nanorod chipset	*-*	S-nr	Input
10	S-nanosheet chipset	*-*	S-nh	Input
11	Acoustofluidic	*-*	*Af*	Input
12	Heat transfer coefficient	*W/(m*^*2*^ *·°C)*	*HTC*	output

### Analyzing the evolution of training curves and impact of hidden layers

**Fig. 2:**
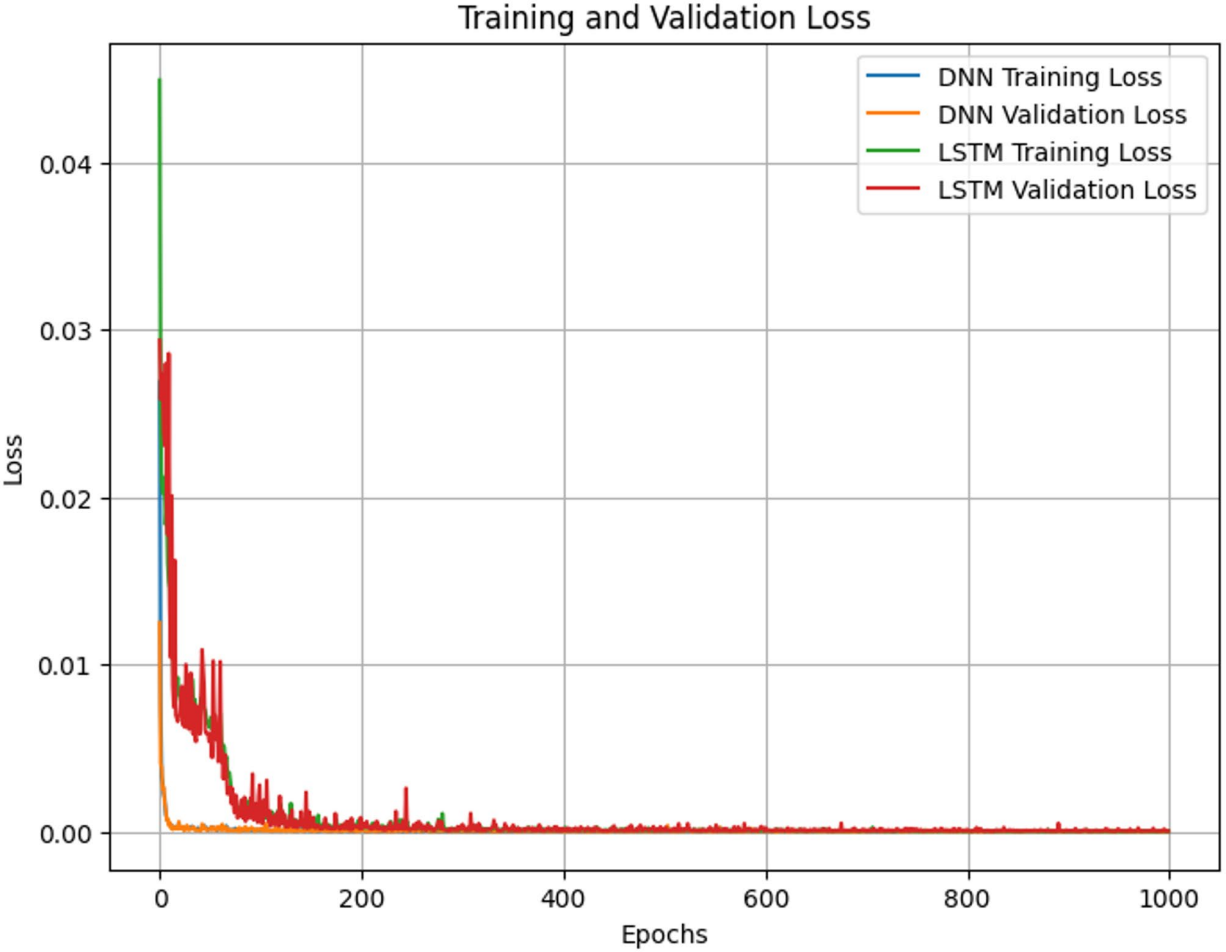
Examining the evolution of training curves in DNN and LSTM.

The capacity of predictive models to learn from data and generalize to new, unforeseen cases is crucial to their success in the field of machine learning. Training loss and validation loss are two crucial metrics for assessing these models’ robustness and performance. In order to determine how well a model fits the training dataset, training loss calculates the difference between the model’s anticipated outputs and the actual labels of the training data. In contrast, validation loss evaluates the model’s performance on a different dataset-often called the validation set-that isn’t utilized during the training stage. The comparison of training loss and validation loss serves as a critical diagnostic tool, helping to identify issues such as overfitting or underfitting, and guiding the optimization process. This study explores the dynamics of training and validation losses, aiming to understand their importance, trends, and implications in model development and evaluation. A deep understanding of these loss metrics is essential for creating machine learning models that perform well on training data and generalize effectively to new, unseen data. Our research examines the factors influencing training and validation losses, including model complexity, dataset size, learning rate, and regularization techniques. The results, presented in Fig. 2, illustrate how these factors interact and affect the two key metrics, demonstrating the importance of fine-tuning models for optimal performance.

**Fig. 3:**
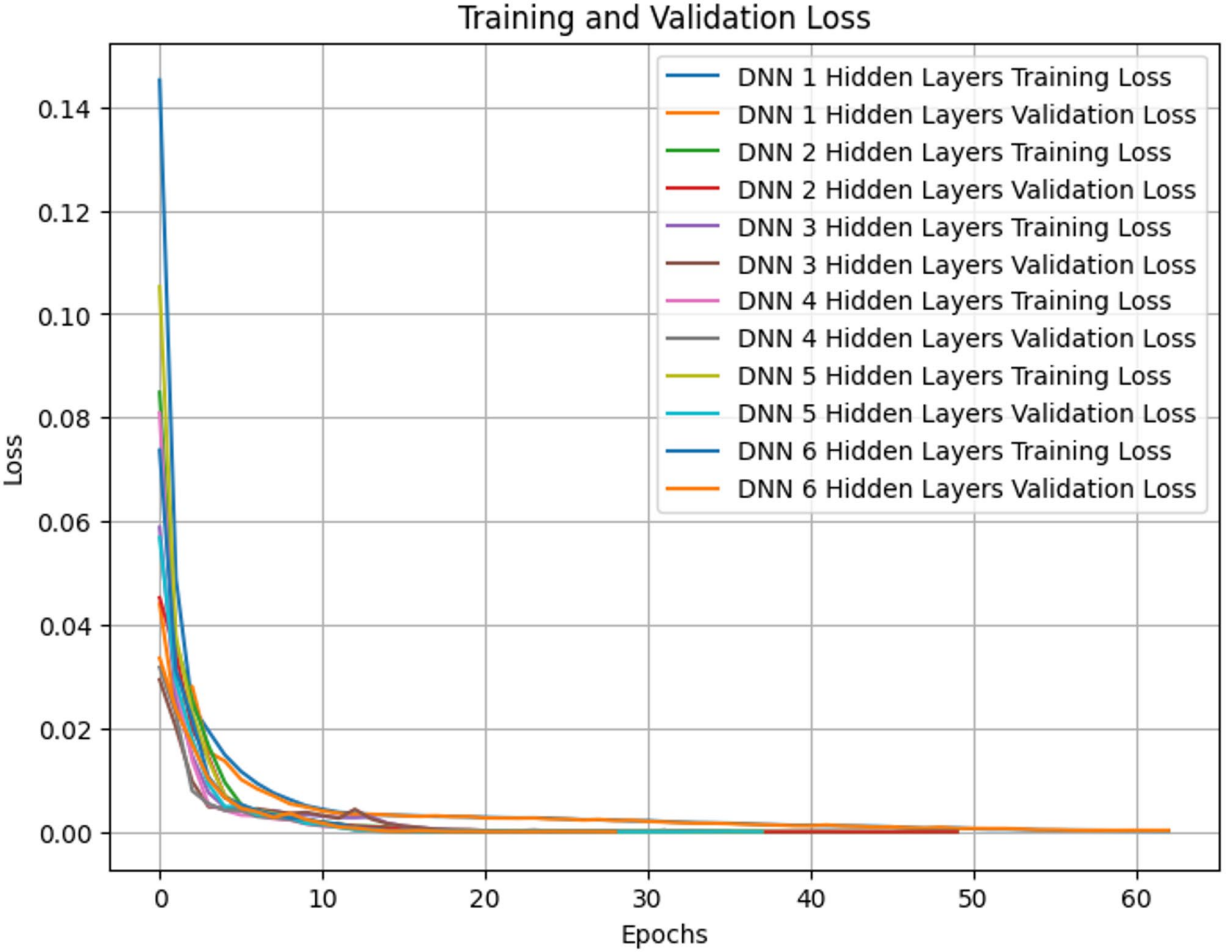
Exploring training loss and validation loss dynamics for unveiling the impact of hidden layers.

**Fig. 4:**
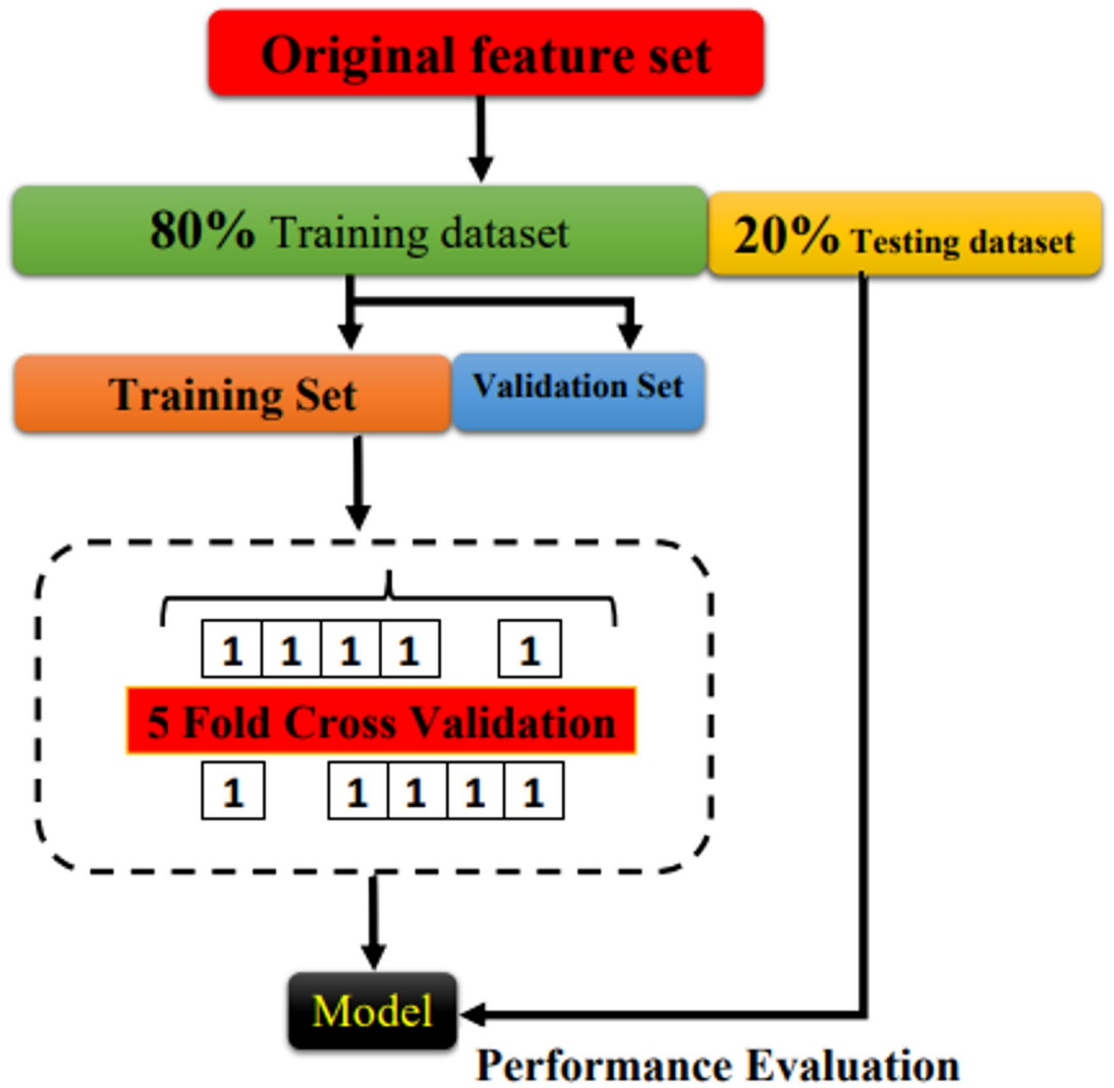
The schematic illustration of the k-fold cross-validation.

As we transition to Fig. 3, our focus shifts towards delving into the correlation between training loss and validation loss concerning the number of hidden layers within the neural network architecture. This investigation aims to discern how altering the depth of the network impacts both the convergence behavior and generalization performance of the model. Hidden layers constitute a pivotal component of neural network architectures, serving as conduits for extracting nuanced features and hierarchical representations from raw input data. However, determining the optimal number of hidden layers is a non-trivial task, influenced by factors such as the complexity of the dataset, the nature of the problem domain, and the computational resources available. In this endeavor, we aim to meticulously analyze the evolving trends of training loss and validation loss across an array of hidden layer configurations. By doing so, we endeavor to unearth insights into how alterations in network depth influence the model’s convergence behavior and its ability to generalize to unseen data. This nuanced understanding is indispensable for striking a delicate balance between the intricacy of the neural network architecture and its efficacy in solving real-world problems. The results gleaned from this analysis, as portrayed in Fig. 4, offer valuable insights into the precise calibration and commendable performance of the models under scrutiny. These findings not only affirm the meticulousness of our model tuning process but also underscore the effectiveness of the optimized neural network architecture in capturing the underlying patterns inherent in the data.

### Comparative analysis of K-fold cross-validation for investigating hidden layers

K-fold cross-validation is a fundamental technique in machine learning that provides a reliable way to carefully examine and strengthen the performance of prediction models. This method involves dividing the original dataset into K folds, or subsets, roughly equal in size^35^. The model then proceeds through K iterations of training, using K-1 folds as the training set and the remaining fold as the validation set. Every fold is used as the validation data exactly once thanks to this iterative approach, which offers a thorough assessment of the model’s performance^36^. K-fold cross-validation’s intrinsic strength is its ability to provide a comprehensive evaluation of model performance, going beyond the constraints of a single train-test split^37^. This approach reduces biases that may result from a particular train-test partition and provides a more reliable evaluation of the model’s generalization ability by combining performance metrics from K validation sets. K-fold cross-validation maximizes the usefulness of existing data by making optimal use of samples for both training and validation tasks, which is especially beneficial in situations with little data^38^. Illustrated in Fig. 4, the schematic structure of a 5-fold cross-validation delineates the sequential steps entailed in training and testing iterations. Each architectural paradigm (LSTM or DNN) exhibits distinct characteristics, excelling in specific datasets while grappling with unique challenges during training and evaluation. A meticulous examination of the data structures encountered by each model type reveals pronounced disparities^39^. LSTM networks are meticulously crafted to navigate sequential data, such as natural language sequences or time series, emphasizing the preservation of temporal dependencies. Conversely, DNNs showcase versatility, accommodating a broad spectrum of data types spanning structured data, images, and text. Consequently, the data partitioning strategies in K-fold cross-validation diverge between LSTM and DNN models^40^. While LSTM models necessitate the preservation of temporal order, DNN models leverage randomization to ensure the creation of diverse training and validation sets. Further discrepancies emerge in the training dynamics and computational requirements. The intricate recurrent connections inherent in LSTM models often demand substantial computational resources and time, particularly when handling lengthy sequences or voluminous textual data. In contrast, DNNs typically exhibit shorter training times, especially for simpler architectures and smaller datasets. Evaluating model performance and discerning overfitting tendencies are pivotal functions facilitated by K-fold cross-validation. For LSTM models, renowned for their adeptness in capturing temporal dependencies, assessing generalization across disparate sections of the data assumes paramount importance. Similarly, DNNs reap substantial benefits from K-fold cross-validation, enabling the identification of overfitting proclivities and gauging their ability to generalize to unseen data. Thus, K-fold cross-validation serves as an indispensable tool, guiding the refinement and optimization of predictive models across a myriad of machine learning applications.

**Fig. 5:**
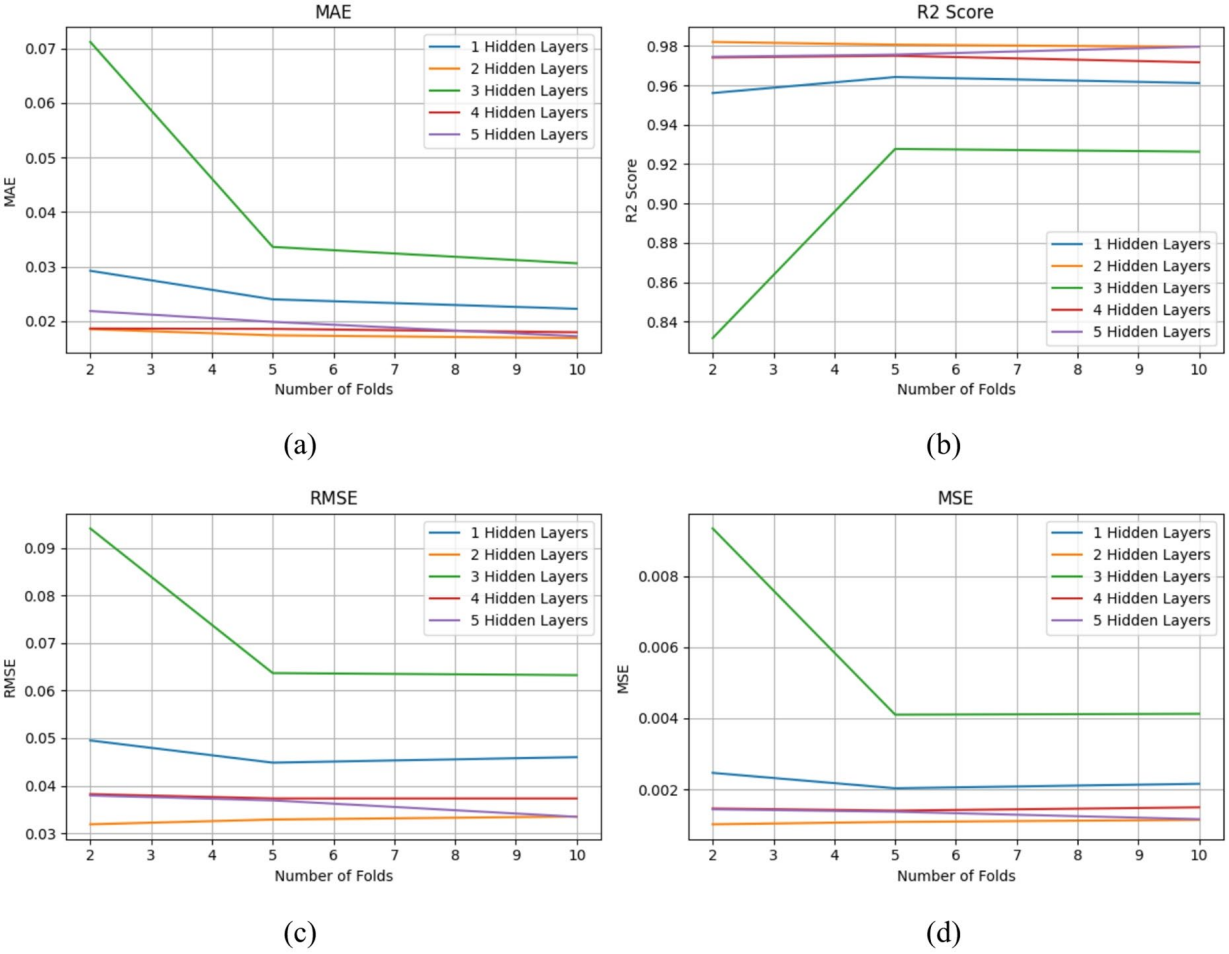
Comparison of K-Fold cross-validation performance across hidden layer configurations: (**a**) MAE, (**b**) R^2^, (**c**) RMSE, (**d**) MSE.

K-fold cross-validation is a reliable method for evaluating how well a neural network can generalize to unseen data. In this study, it was used to systematically test different configurations of hidden layers by dividing the dataset into k parts and repeatedly training and validating the model. This process ensures that the results are not biased by a single train-test split. In the context of hidden layer optimization, the model architecture is systematically varied by adjusting both the number of hidden layers and the number of neurons within each layer. Each configuration is then rigorously assessed using key performance indicators such as accuracy, precision, recall, and error-based metrics including Mean Absolute Error (MAE), Root Mean Square Error (RMSE), and Mean Square Error (MSE). Figure 5 illustrates the comparative evaluation of neural networks with five hidden layers under a 5-fold cross-validation scheme. As shown in Fig. 5a, the MAE results highlight that the 5-fold configuration yields the most stable and optimal performance, with the second hidden layer demonstrating superior predictive capability compared to deeper architectures. Similarly, Fig. 5b examines accuracy trends, where once again, the 5-fold evaluation indicates consistent reliability, and the second hidden layer outperforms networks with a greater number of layers. Figure 5c,d further validate these observations through RMSE and MSE analyses, respectively, both of which confirm that the 5-fold cross-validation approach effectively identifies the second hidden layer as delivering the most balanced trade-off between accuracy and error minimization. Collectively, these findings emphasize the importance of architectural simplicity, where moderately deep models–rather than excessively layered ones–achieve more efficient learning and improved generalization in machine learning-driven optimization frameworks.

### Exploring scaling techniques through density distribution analysis

**Fig. 6:**
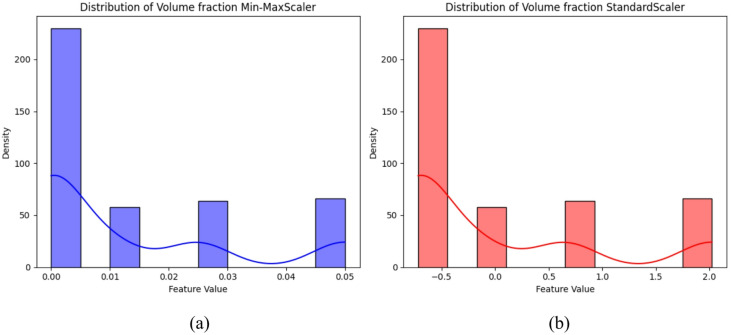
Impact of scaling techniques on density distribution: (**a**) Min-max scaler, (**b**) Standard scaler.

Density distribution analysis proves to be a potent tool in assessing the effectiveness of scaling techniques in ML. This method offers a holistic view of how data distribution transforms pre and post the application of scaling methods, shedding light on their impact on model performance. In our study, we embarked on an exploration of various scaling techniques, including min-max scaling and standardization, to decipher their effects on data distribution (see Fig. 6). Our journey commences with the importation of essential libraries like NumPy, Pandas, and Matplotlib, facilitating data manipulation and visualization. Subsequently, the dataset undergoes preprocessing, encompassing the removal of outliers and missing values to ensure data integrity. Following this, each scaling technique is applied to the dataset, and density distribution analysis ensues on the scaled features. Through density diagrams and histograms, the distribution of features for both scaling models is elucidated. The density distribution plots for the volume fraction employing the Min-Max scaler and the standard scaler exhibit notable disparities in their feature value ranges, despite sharing similar density ranges between 0 and 225 (see Fig. 6a). With the Min-Max scaler, feature values are compressed within a narrow range from 0 to 0.05. This compression is a hallmark of Min-Max scaling, where original feature values undergo linear transformation to fit within a specified range. In this instance, the feature values are normalized to the interval [0, 0.05], retaining the original scaled representation (see Fig. 6b). On the contrary, the density distribution plot for the standard scaler showcases feature values ranging from 0 to 2. Consequently, resulting feature values are centered around zero with a standard deviation of 1, leading to a broader range compared to Min-Max scaling. Considering the obtained results and the unique characteristics of the dataset alongside the requirements of the machine learning algorithm, the Min-Max scale emerges as the preferred choice. Min-Max scaling proves adept at preserving the original distribution while exhibiting sensitivity to outliers, aligning well with the dataset’s nature and the machine learning algorithm’s demands. The Standard Scaling and Min-Max Scaler formulas are expressed as follows:

1 $$\:z=\frac{x-\mu\:}{\sigma\:}$$

2 $$\:{x}_{scaled}=\frac{x-\text{m}\text{i}\text{n}\left(X\right)}{\text{max}\left(X\right)-\text{m}\text{i}\text{n}\left(X\right)}$$

### Optimizing the performance of LSTM and DNN models using hyperparameter tuning

In the field of ML, careful calibration of several variables, or hyperparameters, is essential to get the best model performance. These hyperparameters govern the learning process and directly influence a model’s ability to capture underlying patterns within the data. Understanding and effectively tuning these hyperparameters is crucial for enhancing predictive accuracy and generalization capabilities. Hyperparameters, unlike model parameters which are learned during training, are predefined settings that remain constant throughout the learning process. They encompass a range of configuration options, from learning rates and regularization strengths to architectural choices such as network depth and width in neural networks. Each hyperparameter choice can profoundly impact a model’s performance, making their selection and tuning a critical task in the machine learning pipeline. The quest for optimal hyperparameters often involves a delicate balance. On the one hand, hyperparameters need to be adjusted to promote efficient model convergence and learning. However, it is necessary to prevent overfitting, which occurs when a model performs well on training data but badly on unknown data. It takes a methodical investigation of hyperparameter space using strategies like grid search, random search, and more complex optimization algorithms to reach this equilibrium. Two notable models stand out among the wide variety of neural network architectures: Deep Neural Networks and Long Short-Term Memory Networks. Unlocking the full potential of these models requires an understanding of the critical function and influence of hyperparameters. For example, in LSTMs, the network’s ability to preserve and take advantage of long-range dependencies in sequential data is significantly influenced by hyperparameters such as the number of LSTM units, activation functions for different gates, and sequence length. Similar to this, the architecture and learning dynamics of DNNs are greatly influenced by hyperparameters including activation functions, hidden layer sizes, learning rates, and dropout rates. This investigation explores the complex world of LSTM and DNN hyperparameters, explaining their importance and outlining optimal hyperparameter tweaking techniques. The foundation for managing the fine balance between model complexity and overfitting risk is these careful changes. To properly optimize model performance, these meta-parameters must be adjusted systematically. Through a systematic investigation, the optimization process finds configurations that optimize a model’s performance on unknown data while preserving its effectiveness in the given job. A detailed description of this procedure is given in Table 2, which also outlines the carefully specified parameter values for the two algorithms used, capturing the subtle setups necessary to get the best model performance.

**Table 2:** Best hyperparameters and performance optimization of LSTM and DNN models.

No.	Models	Hyperparameters	
*1*	*DNN*	Dnn-activation	ReLU
		Dnn-alpha	0.1
		Dnn-hidden-layer-sizes	100, 50, 25
		Learning-rate	0.001
		Dropout	0.25
		Embed-dim	150
		Hidden-dim	256
*2*	*LSTM*	Learning-rate	0.001
		Dropout rate	0.4
		Batch size	32
		Optimizer	Adam
		Activation functions	ReLU
		Weight initialization	He initialization
		Sequence length	30

### Investigating hidden layers in the effectiveness of ML algorithms

**Fig. 7:**
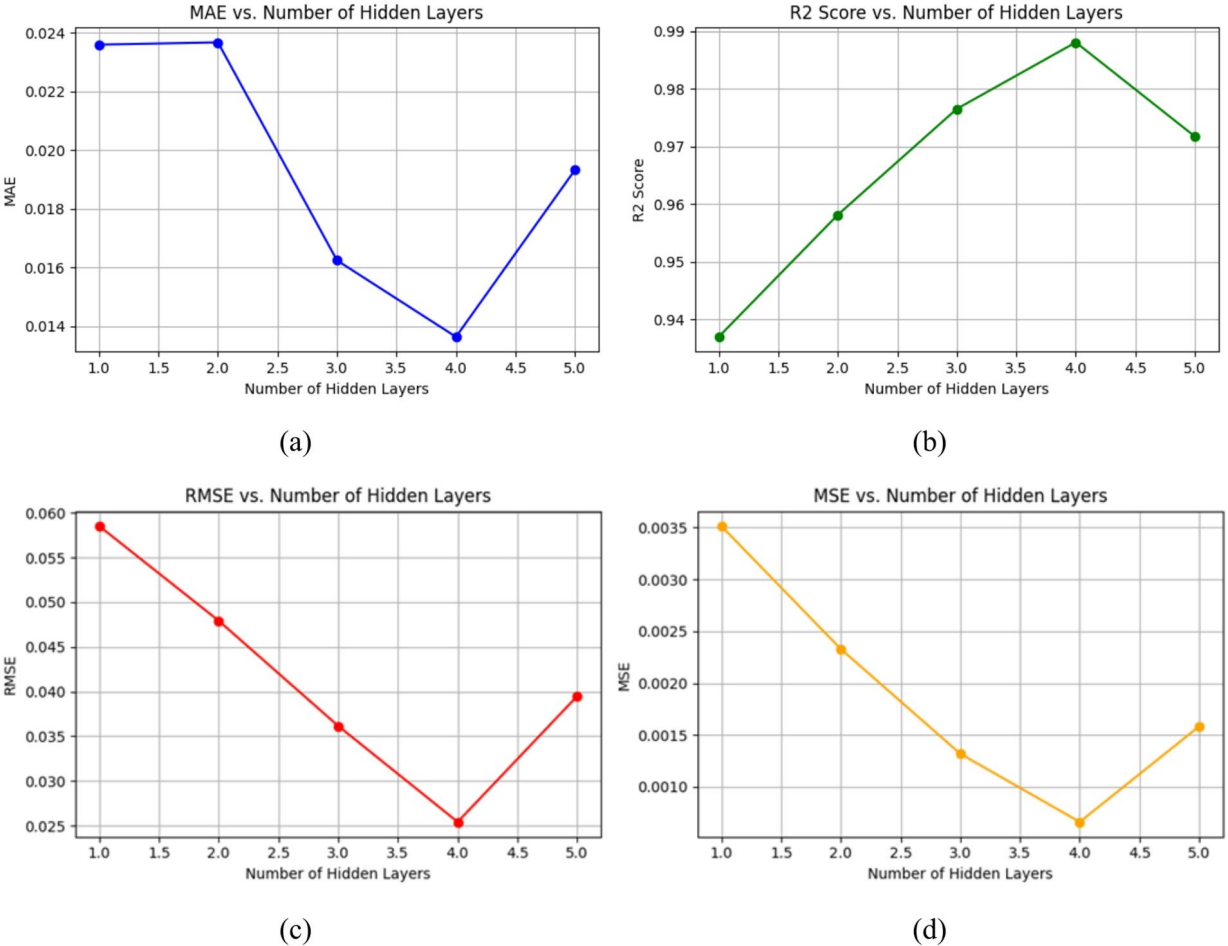
Performance comparison of hidden layer configurations in DNN and LSTM Models: (**a**) MAE, (**b**) R^2^, (**c**) RMSE, (**d**) MSE.

In the dynamic realm of deep learning, the number, size, and choice of activation functions are only a few of the many factors that go into configuring these hidden layers. Each of these components is essential in determining how well the model can identify intricate linkages present in the data. The way hidden layers are strategically arranged within DNNs has a significant impact on the model’s capacity for learning and generalization. The network may gradually abstract characteristics from the input data by increasing the number of hidden layers, which might improve its performance in jobs requiring the identification of subtle patterns. The risk of overfitting is increased by this depth increase, though, therefore a careful balance between generalization and model complexity is required. Similarly, hidden layers are essential to LSTMs because they capture and preserve the temporal relationships present in sequential data. The model’s capacity to identify small temporal patterns and encode them into meaningful representations is directly impacted by the way these layers are configured, including factors like the number of LSTM units and the selection of activation functions. The choice of activation functions is crucial in determining the model’s learning dynamics and convergence behavior, aside from structural factors. Because modified linear units are straightforward and computationally efficient, using them as activation functions makes them ideal for training both DNNs and LSTMs. In order to fully investigate how hidden layers, affect model performance, we conducted a rigorous set of tests in which we varied the number, size, and activation functions of these essential elements in our neural network topologies. We provide a thorough comparison of models with five hidden layers in Fig. 7, highlighting how well they perform on a number of assessment measures, such as MAE, R^2^ score, RMSE, and MSE. A deeper look at Fig. 7a reveals that models with four hidden layers significantly lower the average absolute error, indicating a high degree of agreement with the real experimental data. The efficiency with which these arrangements capture the fundamental patterns seen in the data is demonstrated by this alignment. Interestingly, there is a noticeable tendency towards a progressive increase in the average absolute error as the number of concealed layers rises over 4. This pattern emphasizes the possible trade-off between predictive accuracy and model complexity, suggesting that too complex designs might result in declining performance gains. Figure 7b provides further insight into the performance of models with varying numbers of hidden layers, focusing specifically on their R^2^- scores. Notably, models with 4 hidden layers achieve the highest R^2^ scores, indicating their superior ability to explain the variance in the target variable. This finding underscores the importance of striking a balance between model complexity and interpretability, as excessively complex architectures may lead to overfitting and reduced generalization performance. Furthermore, Fig. 7c,d delve into the models’ predictive accuracy by examining their root mean square error and mean square error values. Once again, models featuring 4 hidden layers emerge as the clear winners, showcasing the lowest RMSE and MSE values among all configurations. This indicates that these models exhibit superior predictive accuracy and are better equipped to minimize the discrepancy between predicted and actual values.

## Results and discussions

### A comparative statistical analysis of DNN and LSTM predictive models

Predictive modeling serves as a fundamental tool across various domains, enabling the utilization of historical data to forecast future outcomes. While regression analysis traditionally dominates this landscape, offering insights into continuous outcome prediction based on independent variables, the focus of this study extends beyond mere model construction. Instead, it delves into a comprehensive evaluation of two prominent ML methodologies: deep neural networks and long short-term memory models. DNNs and LSTMs are renowned for their prowess in capturing intricate patterns and dependencies within data, making them promising candidates for predictive modeling endeavors. To rigorously assess their performance, this research employs a suite of key statistical measures, including MSE, MAE, RMSE, R^2^, Symmetric Mean Absolute Percentage Error (SMAPE), and Explained Variance Score (EVS). These metrics serve as crucial benchmarks for gauging predictive accuracy and the models’ ability to elucidate variance within the data. The findings of this study, meticulously detailed in Table 3, underscore the remarkable performance of both DNN and LSTM models. Notably, both models exhibit significant R-squared values of 0.999 and 0.998, underscoring their proficiency in explaining variance. Furthermore, the LSTM model showcases a remarkably low mean absolute error of 0.0055, surpassing the DNN model and indicating a close alignment between its predictions and experimental observations. However, beyond these quantitative assessments, it becomes evident that both DNN and LSTM models demonstrate commendable robustness in predicting outcomes. This resilience underscores their potential for real-world applications across diverse domains, where accuracy and reliability are paramount. In essence, the study portrays both DNNs and LSTMs as frontrunners in predictive accuracy and performance, showcasing their adaptability and efficiency in navigating complex learning environments.

**Table 3:** Statistical error metrics across ML models.

Models	MAE	RMSE	MSE	EVS	SMAPE (%)	*R*^2^-values
*LSTM*	0.0055	0.0072	5.19 e^−05^	0.999	0.8	0.999
*DNN*	0.00654	0.008	6.4 e^−05^	0.998	1.2	0.998

### An integrated approach to phase-change cooling: micropins, nanoarrays, acoustofluidics, and SHAP analysis

**Fig. 8:**
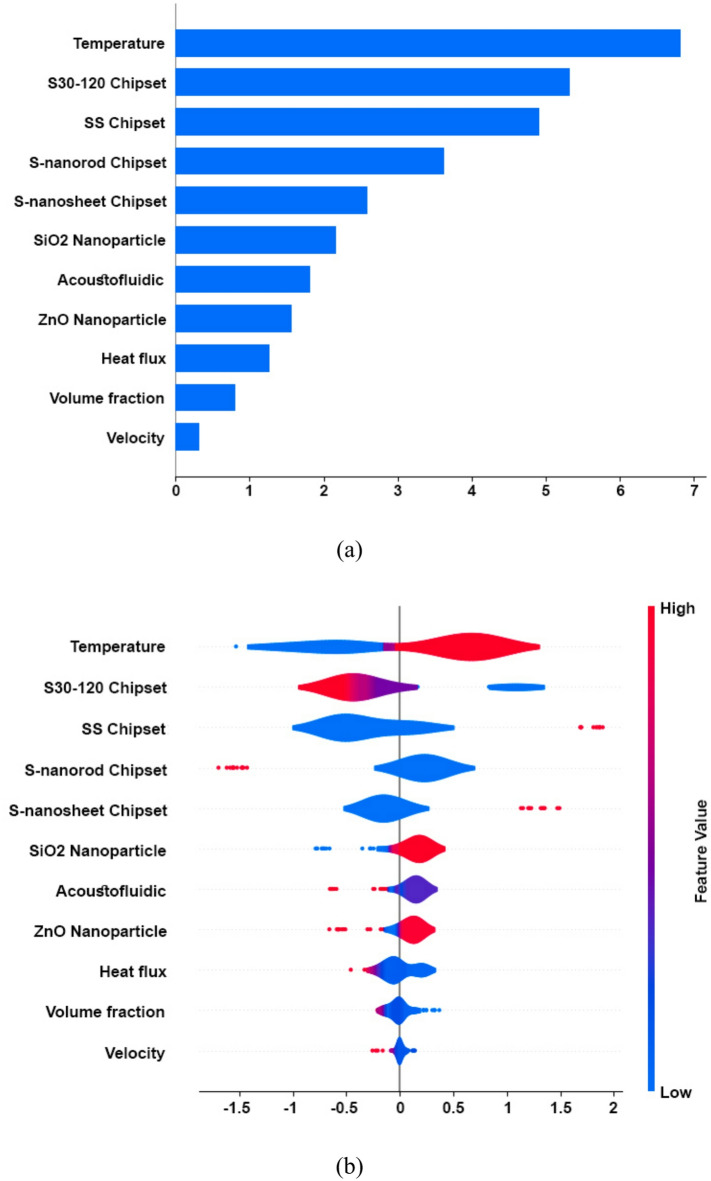
Visualization of SHAP values highlighting influential factors in microfin and micropin structures for enhanced cooling: (**a**) Bar plot, (**b**) Violin plot.

As illustrated in Figure Fig. 8a, our objective is to unravel the intricate relationship among ML, thermal management, and the crucial aspect of interpretability within this multifaceted system. To comprehensively analyze and interpret our experimental findings, we leverage cutting-edge ML moldels, including G-DeepSHAP, DNN, and LSTM. These advanced analytical tools allow us to unravel intricate patterns within our data, providing valuable insights into the underlying factors influencing heat transfer coefficients. Our results highlight how crucial the starting temperature is in determining the system’s heat transmission properties. This observation can be attributed to the central role of temperature in governing thermodynamic properties and phase change dynamics, particularly in two-phase cooling systems employing microfluidic nanofluids or acoustofluidic bubble-based cooling. Variations in initial temperature exert a direct influence on phase change behaviors, with higher temperatures often leading to accelerated phase transitions and subsequent increases in heat transfer rates and HTC values. Furthermore, our investigation sheds light on the temperature-dependent nature of key thermophysical properties of the working fluid, including viscosity, density, and specific heat capacity. These properties undergo significant alterations in response to changes in initial temperature, thereby affecting fluid flow dynamics and convective heat transfer processes within the cooling system. Furthermore, our observations highlight the substantial influence of chipset characteristics on the heat transfer coefficient, emphasizing their critical role in thermal management. Specifically, the S30-120 chipset emerges as a significant determinant of HTC due to its micropin Chip Surface, which incorporates meticulously designed micropin structures aimed at bolstering heat transfer efficiency by maximizing the available surface area for heat dissipation (As illustrated in Fig. 8b). Moreover, the SS chipset serves as a fundamental reference point, influencing HTC in our study with its smooth chip surface devoid of specialized structures like Pin-Fins or nanostructures. This provides essential baseline data for comparative analysis, enabling us to assess the relative impact of surface modifications on HTC. Moreover, the introduction of innovative chipset models, such as the S-Nanorod and S-Nanosheet chipsets, broadens the scope of our investigation. The former integrates or coats micropin structures with ZnO nanorods, while the latter adopts a similar micropin design but utilizes ZnO nanosheets. Leveraging nanotechnology, these advanced chipsets further optimize heat transfer and thermal conductivity, offering promising avenues for enhancing HTC in cooling applications. The choice of chipset surface plays a pivotal role in determining heat transfer efficiency and thermal performance. Specialized structures or coatings, as seen in the aforementioned chipsets, can significantly influence HTC by facilitating improved heat dissipation and phase change processes. In addition to chipset characteristics, the presence of nanoparticles, such as silica nanoparticles, also exerts a notable influence on HTC. Characterized by their high surface-to-volume ratio and surface roughness, silica nanoparticles enhance heat transfer by augmenting the available surface area for heat dissipation, thereby promoting better contact between the fluid and heat source. Furthermore, acoustofluidic techniques emerge as another influential factor in our study, particularly in systems where acoustic waves are harnessed to manipulate fluid flow and heat transfer processes. By leveraging acoustic waves, we can enhance fluid movement, promote phase change phenomena, and optimize heat transfer rates, thereby bolstering HTC in various cooling systems. Additionally, the application of zinc oxide nanoarray coatings on the narrow surfaces of micropin chips represents a cutting-edge approach to enhancing cooling performance. These nanoarrays provide multiple nucleation sites for boiling, facilitating the formation and behavior of vapor bubbles while regulating their nucleation and growth dynamics. Moreover, ZnO nanoarrays exhibit ultrafast liquid rewetting, ensuring continuous and efficient heat transfer by rapidly regenerating the liquid coolant on the chip surface after boiling events. Moreover, beyond chipset characteristics and nanoparticle presence, other key parameters such as heat flux, volume fraction, and velocity also wield significant influence over the HTC in thermal management systems. These parameters play crucial roles in dictating the rate and efficiency of heat transfer processes within cooling systems. Heat flux, representing the rate of heat transfer per unit area, directly impacts the amount of thermal energy exchanged between the cooling medium and the heat source. Similarly, volume fraction, which refers to the proportion of the fluid occupied by particles or other components, influences the overall thermal conductivity and heat dissipation capabilities of the cooling medium. Additionally, velocity, or the speed of fluid flow, affects the convective heat transfer rate by determining how quickly heat is transported away from the heat source.

### Comparative evaluation of prediction and experimental results

**Fig. 9:**
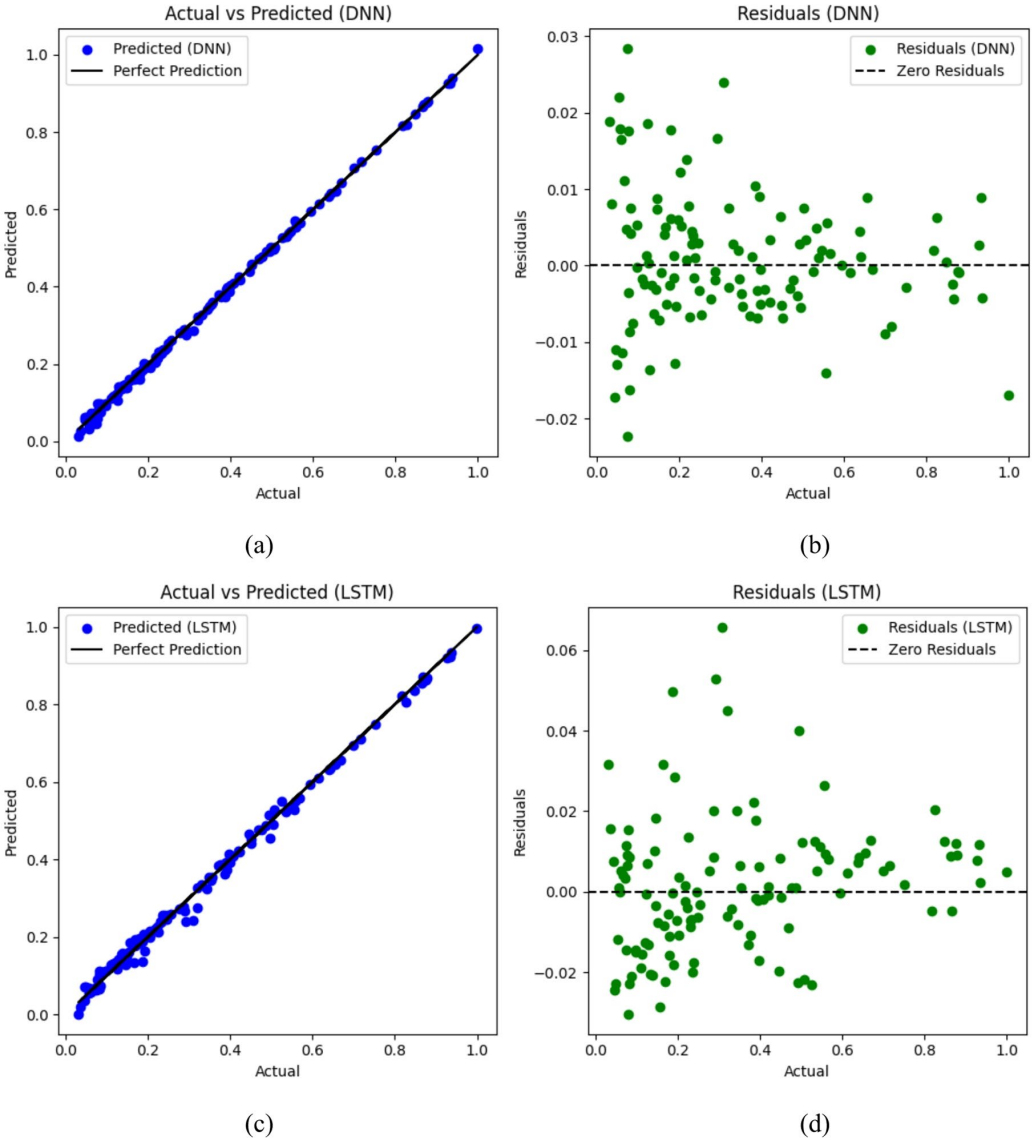
Comparative analysis of experimental and predicted results: (**a**) DNN predictions, (**b**) DNN residuals, (**c**) LSTM predictions, (**d**) LSTM residuals.

In the domain of thermal management, the introduction of high-efficiency two-phase cooling represents a significant departure from traditional heat dissipation methods for a wide array of systems and devices. Unlike conventional cooling techniques like air or liquid cooling, which often struggle to meet rigorous cooling requirements, two-phase cooling presents a transformative solution to thermal regulation challenges. At the heart of a two-phase cooling system lies the exploitation of the phase transition of a working fluid, typically alternating between liquid and vapor states. When heat is generated by electronic components or other sources, it triggers the evaporation of the working fluid, absorbing thermal energy in the process. The resulting vapor then transports the heat away from the heat source to a condenser, where it condenses back into liquid form, releasing the accumulated heat to the environment or an auxiliary cooling system. This cyclical process ensures continuous and efficient heat dissipation, with the liquid recirculated to sustain the cooling cycle. The advantages of two-phase cooling over conventional methods are manifold. It offers heightened heat transfer rates, particularly advantageous in scenarios with high power densities. Additionally, two-phase cooling systems typically occupy less space and weigh less, making them well-suited for constrained environments. Furthermore, their superior thermal performance renders them indispensable in critical applications such as electronics cooling, power electronics, and high-performance computing. Concurrently, the emergence of machine learning has ushered in a new era of innovation across industries, including thermal management. Leveraging ML algorithms enables in-depth analysis of sequential data, providing profound insights for optimizing thermal management processes. Industries, particularly in the electronics sector, have embraced these transformative technologies, integrating ML into their data analysis and predictive modeling workflows. The synergy between ML, and high-efficiency two-phase cooling systems represents a frontier in thermal management technology. By harnessing ML’s cognitive capabilities, these advanced thermal management solutions are poised to redefine heat management across diverse domains. From electronic cooling to energy systems, these cutting-edge solutions promise to elevate performance metrics and drive operational efficiencies to unprecedented levels. In our research endeavor, we embark on a multifaceted exploration aimed at unraveling the complexities of advanced thermal management, with a particular focus on high-efficiency two-phase cooling systems. Leveraging the power of machine learning, specifically deep neural network and long short-term memory models, we aim to predict experimental outcomes relevant to heat exchanger performance. However, our objectives extend beyond prediction; we seek to elucidate the intricate relationships between input variables and the efficacy of thermal management systems. The visual representation provided in Fig. 9 offers a detailed comparative analysis between the predictions generated by our artificial neural network algorithms and the actual experimental data. This comparison serves as a critical validation step, affirming the reliability and efficacy of our machine learning approach in predicting heat transfer coefficients within our thermal management system. The results of our deep neural network approach are displayed in Fig. 9a, which shows an impressive degree of accuracy with a sample accuracy rating of 0.998 and matching absolute errors of 0.0065. This high degree of alignment between predicted and observed values underscores the robustness of our DNN model in capturing the complex relationships inherent in the data. Additionally, Fig. 9b delves deeper into the performance of the DNN model by examining the residuals, which serve as a measure of the model’s ability to adequately represent the underlying data patterns. The consistency demonstrated in the residual analysis further solidifies the reliability of our DNN predictions. Going ahead, our study is expanded to incorporate predictions produced by the long short-term memory model in Fig. 9c. Here, we see a similar degree of accuracy, with the LSTM model obtaining an absolute error of 0.0055 and a sample accuracy rating of 0.999. This meticulous evaluation of both predicted and experimental results reaffirms the robustness and effectiveness of our LSTM model in forecasting HTC values within our thermal management system. Furthermore, Fig. 9d serves as a diagnostic tool, allowing us to identify potential areas for model refinement and optimization to enhance the overall reliability and performance of our LSTM predictions. In conclusion, the findings presented in Fig. 9 underscore the remarkable proficiency of our machine learning models in accurately predicting HTC values within our thermal management system. With an impressive R-squared value of 0.99 achieved by both the DNN and LSTM models, our approach holds tremendous promise for offering invaluable insights aimed at optimizing and enhancing cooling systems across a multitude of applications and industries.

### Partial dependence plot-based insight into nanofluidic–acoustofluidic cooling systems

**Fig. 10:**
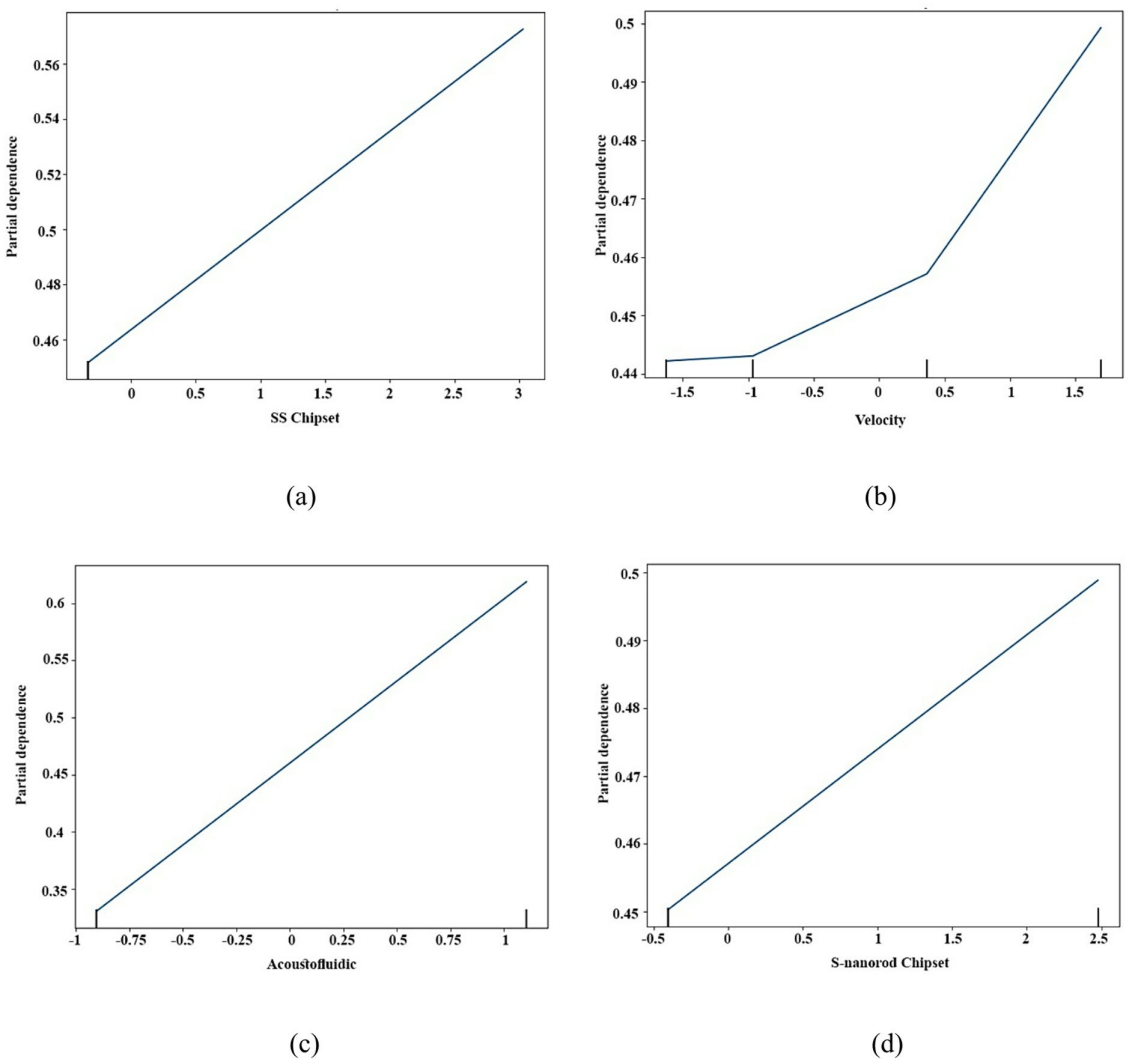
PDP illustrating the marginal effects of acoustic actuation, flow velocity, and chip material on cooling performance: (**a**) SS chipset, (**b**) Flow velocity, (**c**) Acoustofluidic, (**d**) S-nanorod chipset.

The four Partial Dependence Plots (PDPs) in Fig. 10 provide a detailed view of how individual input variables influence the model’s predicted performance, which reflects chip cooling efficiency, thermal conductivity, and bubble-enhanced heat transfer in the microfluidic system. Among the variables, the acoustofluidic parameter (Fig. 10c) exhibits the strongest and most linear positive impact on system performance. The partial dependence curve rises steadily from approximately 0.34 to over 0.66, indicating that increasing the strength of the acoustic field significantly enhances predicted cooling efficiency. This finding highlights the critical role of acoustic excitation in driving advanced heat transport mechanisms within the microchannel environment. Acoustic fields facilitate microbubble formation, induce acoustic streaming, and disrupt laminar boundary layers, thereby enhancing local mixing and convective heat transfer. Such mechanisms are especially vital in two-phase cooling regimes, where phase interface dynamics and fluid-structure interactions are sensitive to external perturbations. These results suggest that acoustofluidic actuation is not only beneficial but essential for achieving high-performance thermal management in compact, high-power-density applications. The stainless steel (SS) chipset (Fig. 10a) also contributes positively to predicted performance. The PDP shows a linear increase from ~ 0.46 to above 0.56, indicating that integrating stainless-steel components enhances thermal efficiency. This effect is consistent with stainless steel’s high thermal conductivity, structural rigidity, and durability at elevated temperatures, which improve heat spreading and reduce localized overheating. Additionally, the SS surface likely supports microbubble transport and acoustic wave propagation, indirectly reinforcing the benefits of acoustofluidic mechanisms. Flow velocity (Fig. 10b) exhibits a nonlinear influence on performance. At low velocities (< 0.5), performance increases gradually, reflecting modest improvements in convective heat transfer under laminar microchannel flow. Beyond a threshold (~ 0.5), the curve steepens, showing accelerated performance gains. This behavior indicates a threshold-dependent mechanism: once inertial forces overcome viscous resistance and thermal boundary layer limitations, convective transport is significantly enhanced. This highlights the importance of flow rate optimization for maximizing heat transfer in nanofluidic systems. Finally, the S-nanorod chipset (Fig. 10d) shows a positive but more moderate effect, with predictions rising from ~ 0.45 to 0.50. This suggests that silicon nanorod structures enhance heat transfer by improving surface wettability, increasing effective surface area, and promoting capillary-driven fluid transport. Although the slope is less steep than for acoustofluidics or flow velocity, these nanoscale enhancements still contribute meaningfully to thermal performance. The moderate impact likely reflects either the secondary role of nanostructures relative to dominant factors or potential saturation effects at higher nanorod densities.

**Fig. 11:**
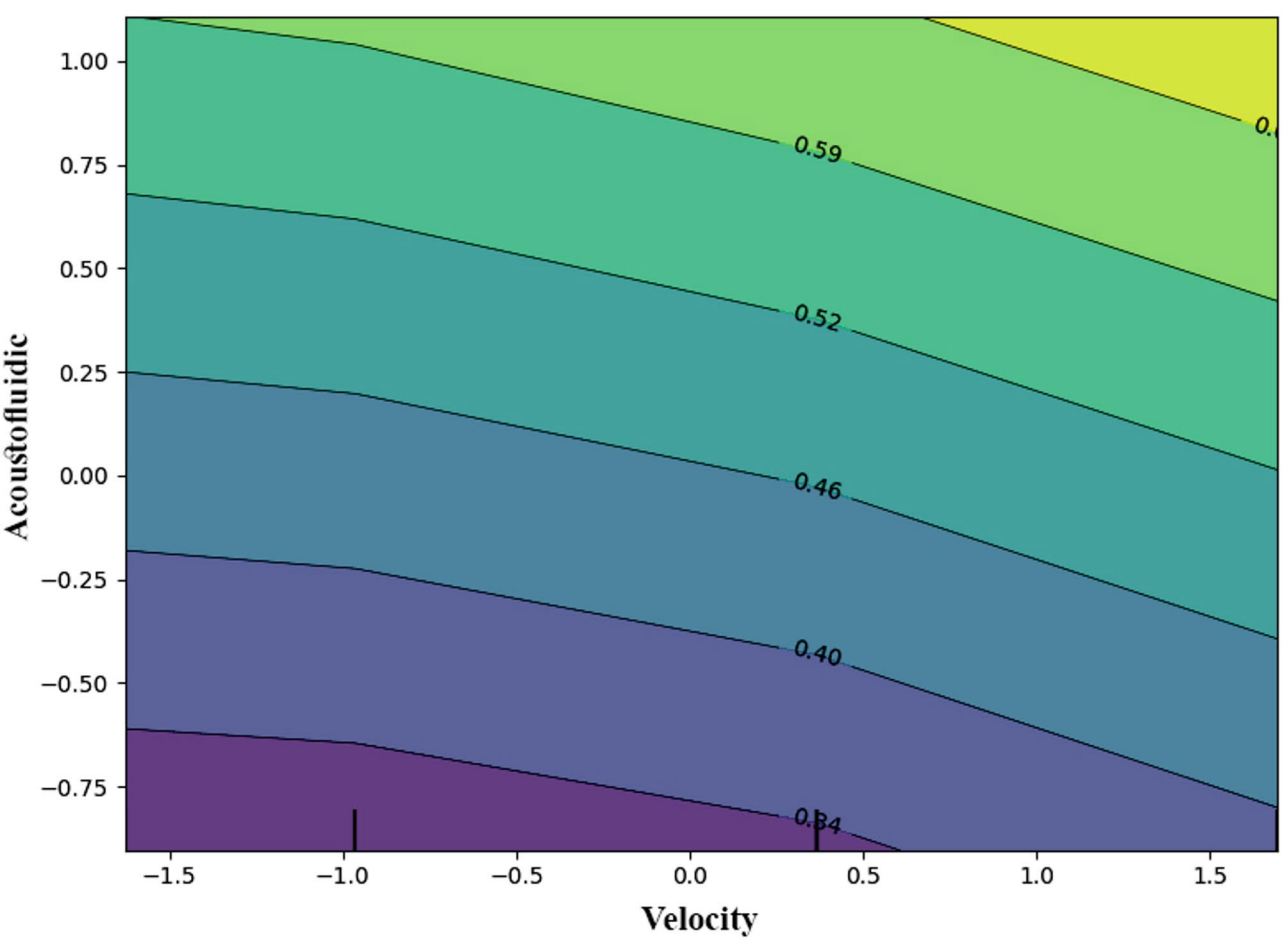
Contour plot showing the combined influence of flow velocity and acoustofluidic field strength on chip cooling performance, highlighting the synergistic region of maximum heat transfer efficiency.

The contour plot presented in Fig. 11 illustrates the dynamic interaction between flow velocity and acoustofluidic activation on a key performance parameter relevant to two-phase microchannel cooling, such as the heat transfer coefficient, bubble stability, or cooling efficiency. The contour gradients in the plot clearly show that increasing either flow velocity or acoustofluidic intensity independently yields improvements in cooling performance. However, the highest performance levels are achieved when both variables are simultaneously maximized, as indicated by the upper-right region of the diagram. This pattern suggests a strong synergistic interaction wherein acoustically induced bubble motion–comprising streaming vortices, boundary layer disruption, and micro-scale turbulence–complements the macroscopic momentum of fluid flow. Such interplay enhances convective mixing, disrupts thermal stratification, and expedites heat removal from the chip surface. Additionally, the smooth, continuous contour lines, without abrupt transitions or inflection points, imply that the relationship between these variables and cooling performance is largely linear or smoothly nonlinear, without abrupt thresholds. This behavior is advantageous from a control systems perspective, as it allows for predictable system responses to incremental adjustments in either parameter. In particular, at lower flow velocities, the role of acoustofluidic stimulation becomes especially prominent. Under such conditions, where laminar flow may dominate and convective heat transport is minimal, acoustic excitation acts as a compensatory mechanism, generating microbubbles or secondary flows that induce local mixing and enhance thermal dispersion. On the other hand, at higher velocities–where convective transport already contributes significantly to heat removal–the relative impact of acoustofluidics diminishes slightly, although it remains beneficial in terms of stabilizing bubble distribution, mitigating coalescence, and maintaining effective two-phase flow regimes.

### Comparative analysis of statistical and SHAP-based feature importance

**Fig. 12:**
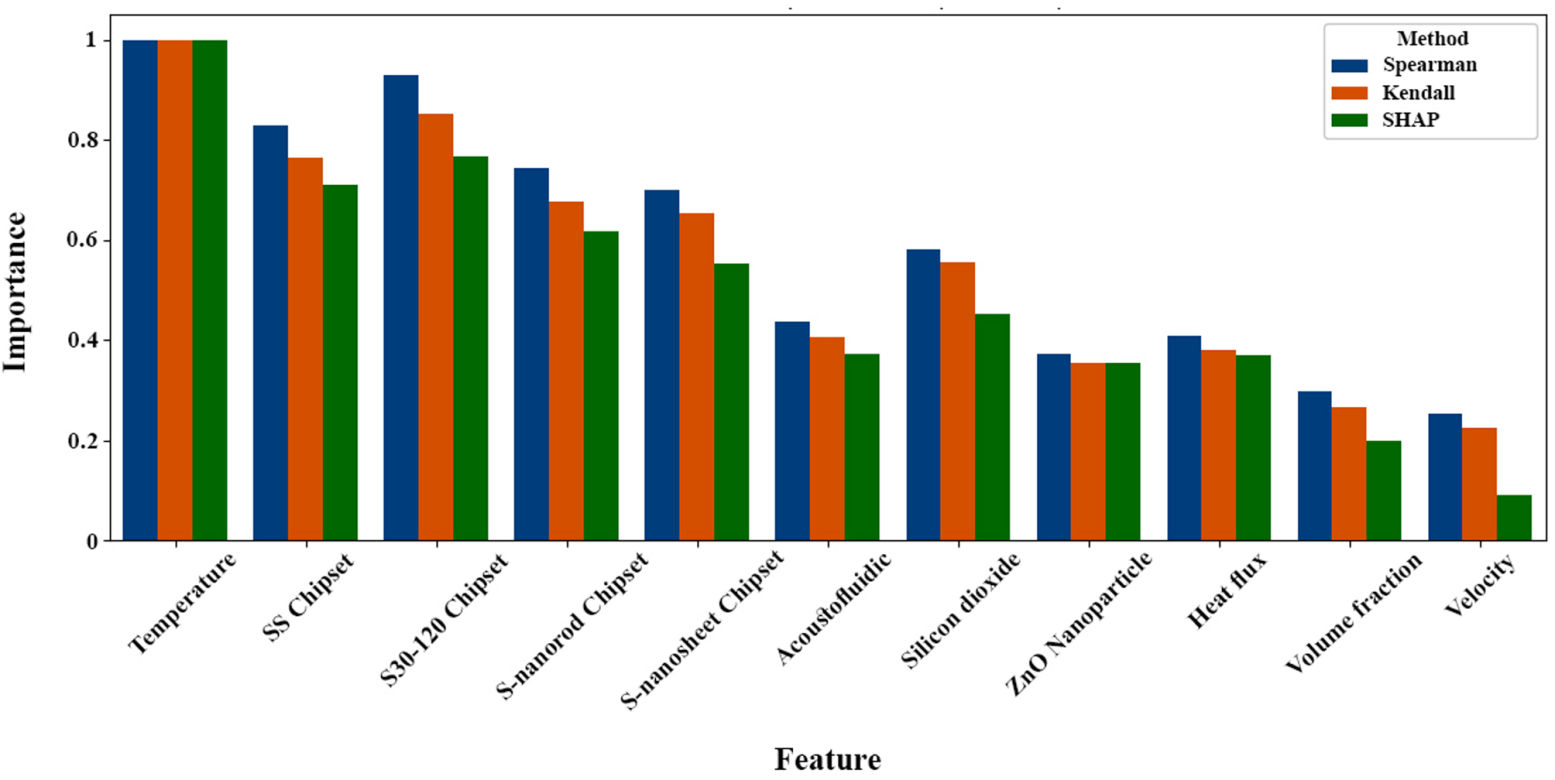
Comparative feature importance rankings for HTC prediction using SHAP, Spearman, and Kendall models analyses.

This analysis is based on experimental data and uses three analytical approaches: Spearman rank correlation, Kendall tau correlation, and SHAP values. The first two methods are statistical techniques that capture the univariate relationships between variables and HTC, while SHAP provides an interpretable framework that quantifies the contribution of each parameter to the model output in a nonlinear and multivariate environment. Based on Fig. 12, the importance of features obtained from the statistical models (Spearman, Kendall), and SHAP for ML-based optimization of two-phase microfluidic cooling systems involving acoustofluidic bubble excitation and nanoarray-coated micropin structures, several critical insights emerge that fundamentally guide our optimization strategy. Temperature emerges as the most important parameter, highlighting its pivotal role as the main driver of phase change processes and heat transfer efficiency, making careful thermal management essential to maximize nucleation sites and bubble dynamics without creating drying conditions. The significant importance of the choice of the chip–specifically the SS chip, the S30-120 chip, the S-nanorod chip, and the S-nanoplate chip–reveals how material properties and nanostructure engineering dramatically affect thermal conductivity, bubble nucleation characteristics, and overall system stability, with each offering distinct advantages in increasing surface area and optimizing the thermal interface. Acoustofluidic activation, as another important factor, confirms our hypothesis that the combination of passive nanostructured surfaces with active acoustic bubble excitation creates powerful synergistic effects that enhance mixing and heat transfer significantly beyond what either approach could achieve independently. The choice of nanoparticles–both silicon dioxide and ZnO–shows moderate but significant importance and influences thermal conductivity changes, surface wettability settings, and bubble formation dynamics, while volume fraction and velocity, although of relatively lesser importance, still require careful optimization to balance thermal enhancement against potential flow resistance effects.

## Conclusions

This study introduces a novel cooling strategy by activating acoustofluidic bubbles, nanoarray-coated micropin structures, and applying statistical and machine learning methods in a two-phase microfluidic platform. Unlike conventional passive cooling approaches, this system actively manipulates the bubble dynamics with ultrasound waves to stabilize boiling, prevent drying, and ensure uniform heat distribution. The use of nanoarray-coated micropins further enhances heat transfer by promoting liquid refilling by capillary conduction and improving surface wettability. In this approach, experimental data are integrated with machine learning models (LSTM and DNN) as well as statistical analyses using Spearman rank and Kendall tau. In addition, the interpretability of the model is achieved through advanced techniques such as SHAP (G-DeepSHAP), PDP. The following main conclusions can be drawing from this investigation:

1. The prediction results show that both LSTM and DNN models achieved an acceptable accuracy rate in predicting the test results, and the LSTM model performed better than the DNN by providing lower MAE (0.0055), SMAPE (0.8), and RMSE (0.0072), indicating better generalizability and quantitative robustness on this dataset.
2. The study’s findings reveal that the initial temperature plays a pivotal role as the predominant factor influencing the heat transfer coefficient. Furthermore, our observations underscore the considerable effects of both the S30-120 and SS chipsets on HTC, underscoring their importance within the domain of thermal management.
3. PDP finding indicates that acoustofluidic actuation has the strongest positive impact on microfluidic cooling performance, followed by stainless-steel chipset design and flow velocity optimization, while nanostructured surfaces provide modest enhancements. The findings highlight the dominance of active acoustic techniques over passive structural modifications in boosting heat transfer. For optimal cooling systems, combining acoustofluidics with optimized flow rates and material selection proves most effective.
4. The contour plot and PDP analysis reveal that coordinated optimization of flow velocity and acoustofluidic activation produces superior cooling performance in microchannel systems. This synergistic combination enhances heat transfer through improved bubble dynamics and fluid mixing while maintaining predictable control characteristics.
5. Based on a multi-method analytical framework integrating statistical correlation techniques (Spearman, Kendall) and ML interpretability (SHAP), this study demonstrates that temperature constitutes the most influential parameter governing HTC performance in two-phase microfluidic cooling systems. The analysis further establishes that chipset selection–specifically SS, S30-120, S-nanorod, and S-nanosheet configurations–serves as a secondary critical factor in thermal performance optimization. Results also indicate that nanoparticle selection (SiO₂ and ZnO) significantly moderates thermal properties and bubble dynamics, while flow velocity and volume fraction require precise calibration to balance thermal enhancement against hydrodynamic resistance.

Building on these promising results, future studies could investigate more advanced machine learning approaches^41^, including graph neural networks, Bayesian Optimization^42^, physics-informed neural networks^43,44^, and ensemble frameworks, to better capture the complex interactions among system parameters. Incorporating real-time adaptive learning could enable dynamic optimization of cooling performance under varying operating conditions. Additionally, research could extend to multi-objective optimization that integrates both thermal and mechanical performance, as well as the exploration of novel nano- and microstructured surface designs to further enhance microfluidic heat transfer.

## Supplementary Information

Below is the link to the electronic supplementary material.

Supplementary Material 1

## Data Availability

The data that supports the findings of this study are available within the article and its supplementary material.

## References

[1] Vasilev, M. P., Abiev, R. S. & Kumar, R. Effect of circular pin-fins geometry and their arrangement on heat transfer performance for laminar flow in microchannel heat sink. *Int. J. Therm. Sci.* **170**, 107177 (2021).

[2] Chen, M., Ji, C., Liu, Z. & Wang, N. Numerical simulation of flow and heat transfer characteristics in Non-Closed Ring-Shaped Micro-Pin-Fin arrays. *Energies 2023*. **16, Page 3481** (16), 3481 (2023).

[3] Ma, X. et al. Saturated/subcooled flow boiling heat transfer inside micro/mini-channels: A new prediction correlation and experiment evaluation. *Int. J. Heat. Mass. Transf.* **210**, 124184 (2023).

[4] Li, H. & Jing, D. Hydraulic and thermal performances of micro pin fin heat sink with increasing pin fin height. *Case Stud. Therm. Eng.* **53**, 103912 (2024).

[5] Ateş, A. et al. Flow dynamics characteristics of flow boiling in minichannels with distributed pin fin structures. *Int. J. Therm. Sci.* **199**, 108912 (2024).

[6] Deng, D., Wan, W., Qin, Y., Zhang, J. & Chu, X. Flow boiling enhancement of structured microchannels with micro pin fins. *Int. J. Heat. Mass. Transf.* **105**, 338–349 (2017).

[7] Mihalko, E. M. & Basak, A. Optimizing thermal performance of pin-fin arrays using bayesian methods for turbine cooling. *Int. J. Heat. Mass. Transf.* **225**, 125355 (2024).

[8] He, W., Wang, Z., Li, J. & Li, Q. Investigation of heat transfer performance for through-silicon via embedded in micro pin fins in 3D integrated chips. *Int. J. Heat. Mass. Transf.* **214**, 124442 (2023).

[9] Ma, X. et al. Jet impingement boiling heat transfer performance of refrigerant HP-1 in micro-pin-finned surfaces for high-power chips. *Int. J. Heat. Mass. Transf.* **221**, 125101 (2024).

[10] Liu, L., Yu, L., Yuan, B., Liu, B. & Wei, J. Flow boiling heat transfer enhancement via micro-pin-fins/ZnO nanorods hierarchical surface. *Int. J. Heat. Mass. Transf.* **203**, 123810 (2023).

[11] Esfahani, I. C., Ji, S., Alamgir Tehrani, N. & Sun, H. An ultrasensitive micropillar-enabled acoustic wave (µPAW) microdevice for real-time viscosity measurement. *Microsyst. Technol.* **29**, 1631–1641 (2023).

[12] Shi, Z., Lan, X., Cao, J., Zhao, N. & Cheng, Y. Numerical study of variable density and height flow guided pin fin in an open microchannel heat sink. *Int. J. Heat. Mass. Transf.* **225**, 125405 (2024).

[13] Esfahani, I. C. & Sun, H. A droplet-based micropillar-enhanced acoustic wave (µPAW) device for viscosity measurement. *Sens. Actuators Phys.* **350**, 114121 (2023).

[14] Lee, Y. J. & Kim, S. J. Experimental investigation on thermal-hydraulic performance of manifold microchannel with pin-fins for ultra-high heat flux cooling. *Int. J. Heat. Mass. Transf.* **224**, 125336 (2024).

[15] Liu, B., Yang, X., Jie, Z., Wei, J. & Li, Q. Enhanced pool boiling on micro-nano composited surfaces with nanostructures on micro-pin-fins. *Int. J. Heat. Mass. Transf.* **190**, 122812 (2022).

[16] Esfahani, Chiniforooshan. I. A data-driven physics-informed neural network for predicting the viscosity of nanofluids. *AIP Adv*10.1063/5.0132846 (2023).

[17] Li, J., Zhang, D., Wang, Y., Zhang, P. & Zhu, G. Flow condensation inside a multiport mini channel and a rectangular mini channel with pin fin array. *Int. J. Heat. Mass. Transf.* **220**, 124954 (2024).

[18] Gu, X., Fan, C. & Sun, Y. Multilevel design strategies of high-performance interfacial solar vapor generation: A state of the Art review. *Chem. Eng. J.* **460**, 141716 (2023).

[19] Fallahtafti, N. et al. Shape optimization of hotspot targeted micro pin fins for heterogeneous integration applications. *Int. J. Heat. Mass. Transf.* **192**, 122897 (2022).

[20] Feng, S., Yan, Y., Li, H., He, Z. & Zhang, L. Temperature Uniformity Enhancement and Flow Characteristics of Embedded Gradient Distribution Micro Pin Fin Arrays Using Dielectric Coolant for Direct Intra-Chip Cooling. *Int J. Heat. Mass. Transf*10.1016/j.ijheatmasstransfer.2020.119675 (2020).33071298

[21] Feng, S., Yan, Y., Li, H., He, Z. & Zhang, L. Reprint of: temperature uniformity enhancement and flow characteristics of embedded gradient distribution micro pin fin arrays using dielectric coolant for direct Intra-Chip cooling. *Int. J. Heat. Mass. Transf.* **161**, 120235 (2020).

[22] Huang, Y. et al. Experimental investigation on flow boiling characteristics of a radial micro pin–fin heat sink for hotspot heat dissipation. *Appl. Therm. Eng.* **219**, 119622 (2023).

[23] Bhandari, P. et al. Design modifications in micro pin fin configuration of microchannel heat sink for single phase liquid flow: A review. *J. Energy Storage*. **66**, 107548 (2023).

[24] Radmard, V. et al. Multi-objective optimization of a chip-attached micro pin fin liquid cooling system. *Appl. Therm. Eng.* **195**, 117187 (2021).

[25] Zhu, G. et al. Transfer learning model to predict flow boiling heat transfer coefficient in mini channels with micro pin fins. *Int. J. Heat. Mass. Transf.* **220**, 125020 (2024).

[26] Wang, D., Wang, D., Hong, F., Zhang, C. & Xu, J. Improved flow boiling performance and temperature uniformity in counter-flow interconnected microchannel heat sink. *Appl Therm. Eng*10.1016/j.applthermaleng.2024.122370 (2024).

[27] Song, J. et al. Inhibition of condensation-induced droplet wetting by nano-hierarchical surfaces. *Chem. Eng. J.* **460**, 141761 (2023).

[28] Borzuei, M. & Baniamerian, Z. Role of nanoparticles on critical heat flux in convective boiling of nanofluids: nanoparticle sedimentation and brownian motion. *Int. J. Heat. Mass. Transf.* **150**, 119299 (2020).

[29] Kalita, S., Sen, D., Sen, P., Das, S. & Saha, B. B. Pool boiling heat transfer enhancement and bubble visualization on a microporous copper over CuO filmed surface through combination of chemical etching and electrochemical deposition. *Int Commun. Heat. Mass. Transf ***144** (2023).

[30] Li, S. et al. Uniformity and stability of droplet formation at T-junctions in symmetrical microchannels. *Chem. Eng. J.*10.1016/j.cej.2024.148718 (2024).38882000

[31] Dai, Y. et al. Dynamic behaviours of monodisperse double emulsion formation in a Tri-axial capillary device. *Micromachines*10.3390/mi13111877 (2022). 36363898 10.3390/mi13111877 PMC9694789

[32] Yao, C., Zhao, Y. & Chen, G. Multiphase processes with ionic liquids in microreactors: hydrodynamics, mass transfer and applications. *Chem. Eng. Sci.* **189**, 340–359 (2018).

[33] Chen, H. et al. Microfluidic production of silica nanofluids for highly efficient two-phase cooling with micro pin-fins structure. *Chem. Eng. J.* **465**, 142799 (2023).

[34] Chen, H. et al. Acoustofluidic bubble-driven assisted functionalized nano-array coated micro pin-fins surface for efficient liquid-vapor phase change chip cooling. *Chem. Eng. J.* **483**, 149101 (2024).

[35] Godasiaei, S. H., Talebizadehsardari, P. & Keshmiri, A. Exploring Alumina Nanoparticle Deposition in Heat Exchangers with Hexagonal Tubes: A Hybrid Approach Integrating Numerical Simulations and Machine Learning. *Results Eng.*10.1016/J.RINENG.2025.106477 (2025).

[36] Godasiaei, S. H., Ejohwomu, O. A., Zhong, H. & Booker, D. Interpretability in Machine Learning for IAQ and HVAC Optimisation: A Response to Oka et al. *Build. Environ.*10.1016/J.BUILDENV.2025.113494 (2025).

[37] Godasiaei, S. H. & Chamkha, A. J. Numerical heat Transfer, part A : applications advancing heat transfer modeling through machine learning : A focus on forced convection with nanoparticles. *Numer. Heat. Transf. Part. Appl.* **0**, 1–23 (2024).

[38] Godasiaei, S. H. & Chamkha, A. J. Exploring novel heat transfer correlations: machine learning insights for molten salt heat exchangers exploring novel heat transfer correlations : machine learning. *Numer. Heat. Transf. Part. Appl.* **0**, 1–18 (2024).

[39] Abriha, D., Srivastava, P. K. & Szabó, S. Smaller is better? Unduly nice accuracy assessments in roof detection using remote sensing data with machine learning and k-fold cross-validation. *Heliyon* **9**, e14045 (2023). 36915546 10.1016/j.heliyon.2023.e14045 PMC10006495

[40] King, R. D., Orhobor, O. I. & Taylor, C. C. Cross-validation is safe to use. *Nat. Mach. Intell.* **3**, 276 (2021).

[41] Anthony, Man Mohammad, Jadidi Amir, Keshmiri Hujun, Yin Yasser, Mahmoudi. Non-unique machine learning mapping in data-driven Reynolds-averaged turbulence models Physics of Fluids 36(9). https://doi.org/10.1063/5.0220444

[42] S. Amirreza, S. Madani Erfan, Vaezi Seyed Sorosh, Mirfasihi Amir, Keshmiri (2025) Predicting flow-blurring droplet size using neural networks and Bayesian optimization: A data-driven approach Machine Learning with Applications 21:100708. https://doi.org/10.1016/j.mlwa.2025.100708

[43] Darioush, Jalili Mohammad, Jadidi Amir, Keshmiri Bhaskar, Chakraborty Anastasios, Georgoulas Yasser, Mahmoudi (2024) Transfer learning through physics-informed neural networks for bubble growth in superheated liquid domains International Journal of Heat and Mass Transfer 232:125940. https://doi.org/10.1016/j.ijheatmasstransfer.2024.125940

[44] Darioush, Jalili Seohee, Jang Mohammad, Jadidi Giovanni, Giustini Amir, Keshmiri Yasser, Mahmoudi (2024) Physics-informed neural networks for heat transfer prediction in two-phase flows International Journal of Heat and Mass Transfer 221:125089. https://doi.org/10.1016/j.ijheatmasstransfer.2023.125089

