# Supplementary material for: ML-Driven optimization of two-phase microfluidic cooling using acoustofluidic bubble actuation and nanoarray-coated micropin structures
Source: Sci Rep. 2025 Nov 17;15:40102. doi: 10.1038/s41598-025-23871-6 (PMC12624137; doi:10.1038/s41598-025-23871-6)
Supplement: Supplementary file 1 — Supplementary Material 1 [file 41598_2025_23871_MOESM1_ESM.pdf]

ML-Driven optimization of two-phase microfluidic cooling using acoustofluidic bubble actuation and nanoarray-coated micropin structures

Seyed Hamed Godasiaei [1*], Pouyan Talebizadehsardari [2*], Amir Keshmiri [3*]

[1] School of Chemical Engineering and Technology, Xi'an Jiaotong University, P.R. China.

[2] Power Electronics, Machines and Control (PEMC) Institute, University of Nottingham, Nottingham, U.K.

[3] School of Engineering, University of Manchester, Manchester, UK

* Corresponding authors

Email: hamedgoodasiay@gmail.com, pouyan.talebizadehsardari2@nottingham.ac.uk (P.T.), A.Keshmiri@manchester.ac.uk

Supporting Information

◆ *Examining Deep Neural Networks in-depth for High-Efficiency Two-Phase Cooling*

Deep neural networks (DNNs), inspired by the complex functioning of the human brain, have become foundational tools in modern artificial intelligence. Rooted in deep learning principles, DNNs consist of multiple layers of interconnected neurons capable of learning hierarchical feature representations from data. These networks iteratively adjust their internal parameters–weights and biases–through training processes such as backpropagation, allowing them to capture complex, non-linear relationships in large datasets. Their strength lies in the ability to learn patterns autonomously, enabling high performance across tasks such as image and speech recognition, natural language understanding, and predictive analytics. A standard DNN architecture includes three key components: an input layer for receiving data, one or more hidden layers where most computations occur, and an output layer that generates predictions. Each inter-neuron connection is assigned a weight, while each neuron has a bias value that controls its activation. The feedforward process moves input data through the layers, applying nonlinear transformations at each stage. The model then compares its predictions to ground truth values and updates its parameters using backpropagation to minimize error.

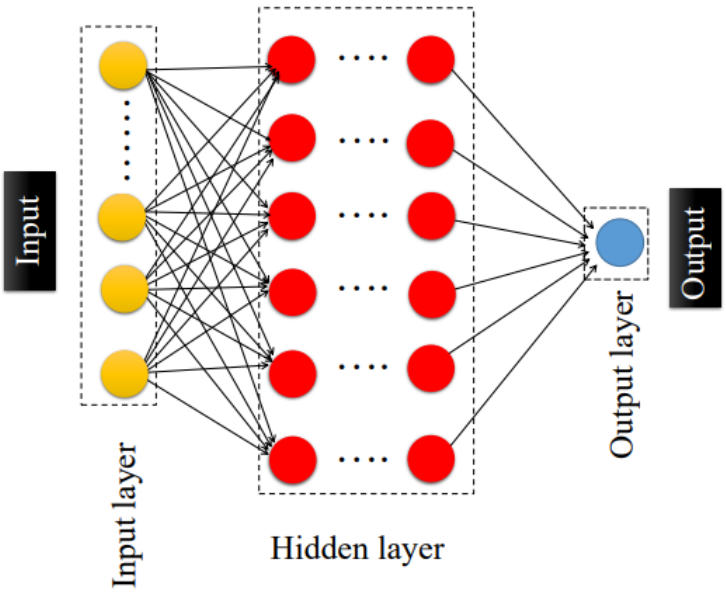

Figure 1: Diagrammatic representation of the High-Efficiency Two-Phase Cooling DNN tree model

◆ *A Comprehensive Exploration of Long Short-Term Memory Networks*

Examining the intricacies of sequential data becomes a crucial endeavor with wide-ranging consequences in the fields of artificial intelligence and machine learning. As artificial intelligence continues to advance in various industries, the electronics sector has enthusiastically embraced these developments. The integration of artificial intelligence into data analysis and predictive modeling has greatly enhanced the industry, offering new insights and efficiencies. In this landscape, high-efficiency two-phase cooling and boiling systems are at the forefront of thermal management technology. Leveraging artificial intelligence capabilities, these advanced systems are committed to improving performance and efficiency, thereby transforming heat management and heat dissipation across various fields. Nevertheless, amid these advances, traditional neural architectures, especially recurrent neural networks, face significant challenges when dealing with complex long-range dependencies inherent in sequential data. While RNNs have proven adept at capturing sequential patterns and dynamics, they struggle to detect subtle relationships across broad temporal contexts. The main obstacle facing RNNs is the vanishing gradient problem, where the gradients decrease exponentially over time, hampering the network's ability to retain information over large sequences. As a result, RNNs face difficulties in maintaining context and providing accurate predictions, especially in scenarios that require assimilation of long temporal relationships. In response to these challenges, Long Short-Term Memory (LSTM) networks have emerged as a transformative neural architecture designed to overcome the limitations of conventional RNNs. At the core of LSTM's innovation lies its complex memory cell structure, which is equipped with specialized gate mechanisms that regulate the flow of information. Unlike the linear information propagation seen in traditional RNNs, LSTMs have gates that exert selective control over the flow of information and enable the network to prioritize relevant information. At the center of the LSTM cell are three key components: the forgetting gate, the input gate, and the output gate. These components, built on sigmoid and '*tanh'* activation functions, work together to manage the internal state of the cell and direct the propagation of information throughout the network. A visualization of the short-term memory network is shown by Figure 2 to give a more realistic perspective. The forget gate evaluates the state of the previous cell in relation to the current state and determines which information should be retained and which should be discarded. Using the sigmoid activation function, the forgetting gate generates a vector that intelligently adjusts the state amplitude of the anterior cell. Simultaneously, the input gate monitors the assimilation of

new insights into the state of the cell and coordinates the selective influx of relevant information while generating new candidate values. By aligning cell state evolution with input gate decisions, LSTM skillfully directs information flow and ensures textual coherence among fluctuating data. Conversely, the output gate determines the relationship of various aspects of the cell's state to the current output. Using a sigmoid activation function, the output gate adjusts the exposure of the current cell state to the next hidden state, ensuring that focus remains on salient insights while filtering out extraneous details. By integrating these triggering mechanisms, LSTMs facilitate the efficient capture of long-term dependencies in sequential data, making them suitable for a range of tasks, including natural language processing, speech recognition, sentiment analysis, and temporal prediction. In addition, LSTMs have sparked a wave of innovations and adaptations, including bipartite LSTMs, cumulative LSTMs, and attention-based mechanisms, which have caused a renaissance in the realm of complex sequential data analysis. The update equations governing LSTM units form a foundational framework for understanding the intricate dynamics that drive information processing and retention within LSTM networks.

$$h^{(t)} = g_0^{(t)} f_h\left(s^{(t)}\right) \tag{1}$$

$$s^{(t-1)} = g_f^{(t)} s^{(t-1)} + g_i^{(t)} f_s\left(wh^{(t-1)}\right) + ux^{(t)} + b \tag{2}$$

$$g_i^{(t)} = sigmoid\left(w_i h^{(t-1)} + u_i x^{(t)} + b_i\right) \tag{3}$$

$$g_f^{(t)} = sigmoid\left(w_f h^{(t-1)} + u_f x^{(t)} + b_f\right) \tag{4}$$

$$g_o^{(t)} = sigmoid\left(w_o h^{(t-1)} + u_o x^{(t)} + b_o\right) \tag{5}$$

Equations 1 to 5, which govern the operation of LSTM units, are frequently referenced in scholarly discussions as a detailed exposition of the mechanisms driving information flow within the network. The network can recognize and preserve long-term dependencies that are typical of sequential data thanks to these equations, which describe how the LSTM cell selectively integrates, updates, and propagates inputs across temporal domains. Depending on the particular implementation and structural subtleties of the LSTM architecture, which includes important functions like input modulation, memory updates, and gating mechanisms, its exact formulation may vary. These equations together capture the core of how LSTM networks use mathematical

operations and activation functions to encode temporal patterns and traverse the complex structure of sequential input.

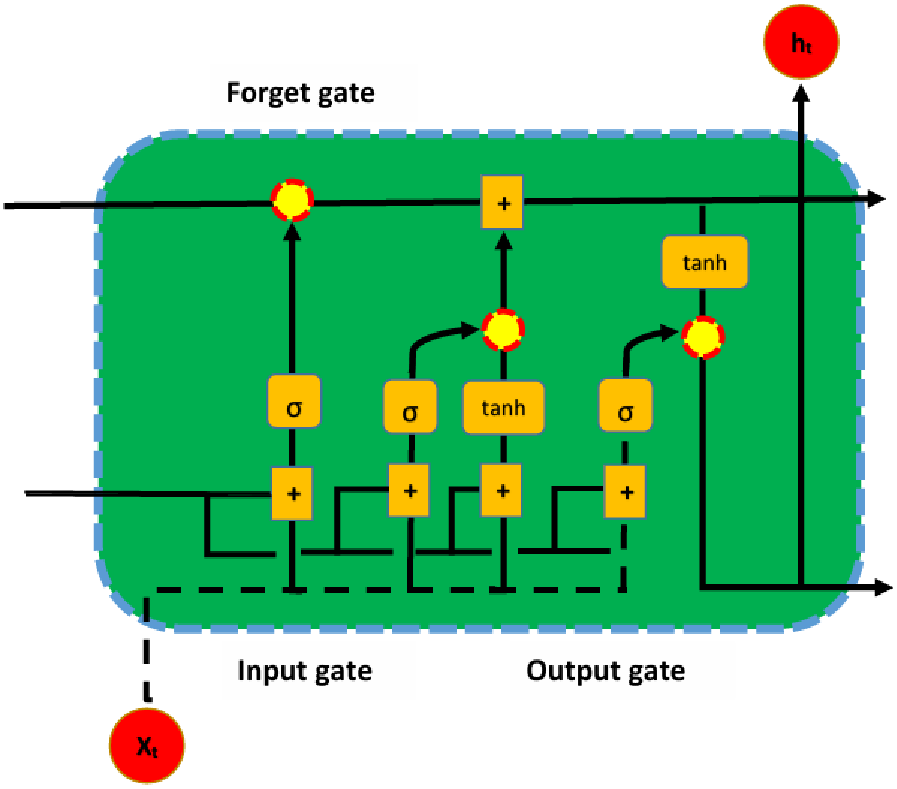

Figure 2: A Schematic Illustration of LSTM Structure

◆ *Utilizing Fundamental Statistical Metrics for Precision Assessment in ML*

In the ever-evolving fields of data science and ML, assessing model performance is essential to developing reliable and strong predictive analytics. Metrics carefully crafted to measure the effectiveness and accuracy of models in identifying and predicting underlying patterns within datasets are at the core of this effort. Among these metrics, the MAE, MSE, RMSE, and R^2-value emerge as indispensable tools, offering nuanced insights into model performance across various applications. RMSE, MAE, MSE, and R^2 represent pillars in model evaluation across a spectrum of programs and applications. These metrics encapsulate distinct facets of model accuracy, providing comprehensive assessments of predictive performance. MAE illuminates the average deviation between predicted and actual values, offering a clear indication of a model's precision. Conversely, RMSE delves deeper, quantifying the spread of residuals and offering insights into

the magnitude of prediction errors. Meanwhile, MSE scrutinizes squared errors, furnishing vital information for optimization algorithms and highlighting the influence of outliers on model fidelity. Complementing these measures, the R-squared value serves as a beacon of model fit, elucidating the extent to which the variance in the dependent variable is explained by the independent variables. In the pursuit of accuracy, model evaluation demands a systematic and rigorous approach, leveraging these metrics to glean multifaceted insights into predictive performance. Each metric unveils a unique vantage point, enabling us to navigate the intricacies of model assessment with clarity and precision. Their mathematical formulations are as follows:

$$R^2 = 1 - \frac{\sum_{i=1}^{n}(y_i - \hat{y}_i)^2}{\sum_{i=1}^{n}(y_i - \bar{y})^2} \tag{6}$$

The R^2-value is a crucial indicator that provides valuable information about how well the model describes the variation in the observed data. On a scale of 0 to 1, a higher R^2 value indicates a greater ability to explain the data, highlighting the model's ability to identify and uncover hidden patterns in the data.

$$MSE = \frac{1}{n}\sum_{i=1}^{n}(\hat{y}_i - y_i)^2 \tag{7}$$

$$RMSE = \sqrt{\frac{1}{n}\sum_{i=1}^{n}(\hat{y}_i - y_i)^2} \tag{8}$$

RMSE stands for the square root of the mean squared error and is used as a supplement to MSE. This metric gives a sense of the typical amount of prediction mistakes by evaluating the average magnitude of errors. Higher model accuracy and precision are shown by lower RMSE values.

$$MAE = \frac{1}{n}\sum_{i=1}^{n}|\hat{y}_i - y_i| \tag{9}$$

MAE provides a straightforward evaluation of the model's overall accuracy by computing the MAE between predicted and observed values. Better performance is shown by reduced MAE values, especially when it comes to reducing absolute prediction mistakes.
